# Database of cattle candidate genes and genetic markers for milk production and mastitis

**DOI:** 10.1111/j.1365-2052.2009.01921.x

**Published:** 2009-12

**Authors:** J Ogorevc, T Kunej, A Razpet, P Dovc

**Affiliations:** Department of Animal Science, Biotechnical Faculty, University of LjubljanaDomzale, Slovenia

**Keywords:** association study, candidate genes, gene linkage, knockout models, mammary gland, mastitis, methylation, micro RNA, milk traits, quantitative trait loci

## Abstract

A cattle database of candidate genes and genetic markers for milk production and mastitis has been developed to provide an integrated research tool incorporating different types of information supporting a genomic approach to study lactation, udder development and health. The database contains 943 genes and genetic markers involved in mammary gland development and function, representing candidates for further functional studies. The candidate loci were drawn on a genetic map to reveal positional overlaps. For identification of candidate loci, data from seven different research approaches were exploited: (i) gene knockouts or transgenes in mice that result in specific phenotypes associated with mammary gland (143 loci); (ii) cattle QTL for milk production (344) and mastitis related traits (71); (iii) loci with sequence variations that show specific allele-phenotype interactions associated with milk production (24) or mastitis (10) in cattle; (iv) genes with expression profiles associated with milk production (207) or mastitis (107) in cattle or mouse; (v) cattle milk protein genes that exist in different genetic variants (9); (vi) miRNAs expressed in bovine mammary gland (32) and (vii) epigenetically regulated cattle genes associated with mammary gland function (1). Fourty-four genes found by multiple independent analyses were suggested as the most promising candidates and were further *in silico* analysed for expression levels in lactating mammary gland, genetic variability and top biological functions in functional networks. A miRNA target search for mammary gland expressed miRNAs identified 359 putative binding sites in 3′UTRs of candidate genes.

## Introduction

Association and quantitative trait locus (QTL) studies in large farm animals are typically performed in outbred populations, making the identification of robust QTL and candidate genes difficult and less reliable due to the variation of genetic background and population-specific interactions between loci. This situation differs very much from the situation in model and laboratory animal species, where highly inbred lines and targeted gene knock-outs are available. Therefore, the only applicable approach for QTL identification and candidate gene detection in large farm animals is the combination of different pieces of evidence supporting the functionality of identified genomic regions in relation to multigenic traits (Mackay 2004). Guidelines and standards for reporting quantitative trait nucleotide discovery in livestock species which allow incorporation of QTL in breeding programmes have been reviewed by Ron and Weller 2007.

A fair amount of genetic research related to lactation and udder health has already been performed due to its economic importance for milk production and manufacturing. This has led to considerable improvement of milk yield (MY); however, the progress in technological properties of milk and udder health has been relatively slow. Shook (2006) reported that somatic cell score (SCS) associated loci have been proposed to improve resistance to mastitis in dairy cattle. In addition, the expression of micro RNAs (miRNAs) in the bovine mammary gland could also play an important role in regulatory pathways in mammary gland development, milk production and resistance or susceptibility to mastitis (Silveri *et al.* 2006).

The recent developments in molecular biology have opened the possibility of exploiting heterologous animal models for comparative studies (Shook 2006). Targeted gene disruption in mice (gene knock-out experiments; KOs) revealed several mammary gland related phenotypes. The release of cattle genome sequence has enabled discovery of new markers and creation of synteny maps including data from other species. For example, Ron *et al.* (2007) utilized murine gene expression data from multiple analyses combined with bovine QTL mapping data to identify candidate genes for QTL for milk production traits in dairy cattle.

Functional traits of the mammary gland have been studied using different approaches, including the QTL approach, association studies and the candidate gene approach. However, information extracted from these methodologically focused studies is fragmented and often controversial. Therefore, there is an urgent need to integrate information from different sources and to allow complementation of different pieces of evidence based on holistic, map driven approach. The possibility of searching the database using animal trait ontology terms to select targets based on the mapping information or to search for indicated sequence similarities in primary databases opens up the possibility of introducing complex decision-making strategies which integrate multiple pieces of evidence supporting the candidate status of the selected region.

The classical forward genetics approaches which are typically focused on a single gene effect have been successful in the identification of a limited number of causal genes. In dairy cattle, two genes, *DGAT1* (Grisart *et al.* 2002) and *ABCG2* (Cohen-Zinder *et al.* 2005), have been reported to affect MY and milk composition. Therefore, the identification of key drivers related to complex traits needs a more holistic approach, based on integration of gene-to-gene interactions with DNA variation data. This approach has recently been developed to elucidate the complexity of common human diseases by intersecting genotypic, molecular profiling and clinical data in segregating populations (Schadt 2006).

Our attempt was to create a database which would take advantage of a multidisciplinary approach linking different types of data and supporting the evidence for involvement of candidate loci into the mammary gland development, milk production traits and resistance or susceptibility to mastitis. The database aims to serve as a tool for systematic development of markers for potential use in marker-assisted selection (MAS), which could be used in cattle breeding programmes to address the most relevant physiological pathways in the mammary gland.

## Materials and methods

The database contains candidate loci involved in mammary gland development, milk production and resistance or susceptibility to mastitis. Candidate loci were collected considering seven different research approaches: (i) gene knock-outs and transgenes in mice that result in specific phenotypes associated with mammary gland; (ii) cattle QTL for milk production and mastitis traits; (iii) loci with sequence variations that show specific allele-phenotype interactions associated with milk production or mastitis in cattle; (iv) genes with expression profiles associated with milk production or mastitis in cattle or mouse; (v) cattle milk protein genes that exist in different genetic variants; (vi) miRNAs expressed in bovine mammary gland; (vii) epigenetically regulated cattle genes associated with mammary gland function.

### Data mining and description of the database

We reviewed the literature published up to December 2008 searching for the relevant publications through PubMed (http://www.ncbi.nlm.nih.gov/pubmed/) and Web of Science (http://isiknowledge.com) using key phrases: genetics, gene candidates, mammary gland, miRNA, mastitis, milk, epigenetics, methylation, QTL, SNP, association. The data from animal experiments were retrieved from the Mouse Genome Informatics (MGI) database (http://www.informatics.jax.org) using the phenotype ontology terms listed in Table S1, representing ontology terms revealed by the literature review.

Quantitaive trait loci were extracted from Cattle QTL Database Release 7 (1/2009): http://www.animalgenome.org using ontology terms associated with mastitis [SCS, somatic cell count (SCC), clinical mastitis (CM)] and milk traits [MY, milking speed (MSPD), dairy capacity composite index (DCCI), protein yield (PY), protein percentage (PP), protein content (PC), energy yield (EY), fat percentage (FP), fat yield (FY), fat content (FC)]. Candidate genes from expression experiments for QTL for milk production traits in cattle were retrieved from cgQTL database (http://cowry.agri.huji.ac.il/QTLMAP/qtlmap.htm).

Putative target sites for mammary gland expressed miRNAs in candidate genes were obtained using Sanger’s mirBase Targets – Version 5 (http://microrna.sanger.ac.uk/). Ensembl transcript identifiers for candidate genes were obtained from Ensembl database – Release 52 (http://www.ensembl.org/) and matched to the list of identifiers with putative miRNA target sites for miRNAs experimentally confirmed in the mammary gland. Polymorphisms in bovine miRNA target octamers of candidate genes were obtained from the Patrocles database (http://www.patrocles.org/).

Candidate genes identified in multiple studies (using the same or different approaches) were considered as the most promising candidates and were analysed for expression level in lactating mammary gland using GNF BioGPS (http://biogps.gnf.org), considering mouse expression data (data for *Bos taurus* are not available yet). Gene variation data of the most promising candidate genes in the promoter region (5 kb), 5′UTR, exon, intron (100 bp flanking sequence) and 3′UTR were obtained from Ensembl database (http://www.ensembl.org/). The ingenuity pathway analysis program (http://www.ingenuity.com) was used to cluster the most promising candidate genes in functional networks.

Our database was created in the Excel format and is available on-line: http://www.bfro.uni-lj.si/Kat_genet/genetika/mammary_gland.xls. Each gene from the mouse KO and gene transfer experiments is hyperlinked to phenotypic allele details in MGI database. Each QTL is hyperlinked to details in Cattle QTL database. The miRNAs are hyperlinked to details in the Sanger miRBase (http://microrna.sanger.ac.uk/) for miRNAs available in the database. Each gene from expression and association studies is hyperlinked to the Map Viewer –*Bos taurus* build (4.0) on NCBI (http://www.ncbi.nlm.nih.gov) or to MGI’s gene details, in cases when gene position for cattle was not available in the Map Viewer. Selected candidate genes and genomic loci were drawn on the genetic marker map.

### Defining the map locations of the loci

The map location was retrieved from NCBI database *Bos taurus* build (4.0). If the map location was not available, we identified the location of the locus using the bovine–human synteny map. The bovine–human synteny map was constructed through BLASTing 8294 markers from MARC and RH maps (Everts-van der Wind *et al.* 2004; Itoh *et al.* 2005) with bovine contigs to obtain hits (defined as *E* < 10^−19^) with longer sequences. Hits were further BLASTed against the human genome; 6231 putative human bovine orthologs were found. Positions on the human physical map were obtained using Map Viewer on NCBI. The syntheny map was constructed using 6023 orthologs sorted in 213 blocks of synteny. Each synteny block with at least two markers (singletons were excluded) is described by its position on the physical human map and on the bovine cytogenetic map.

## Results

**Figure 1:**
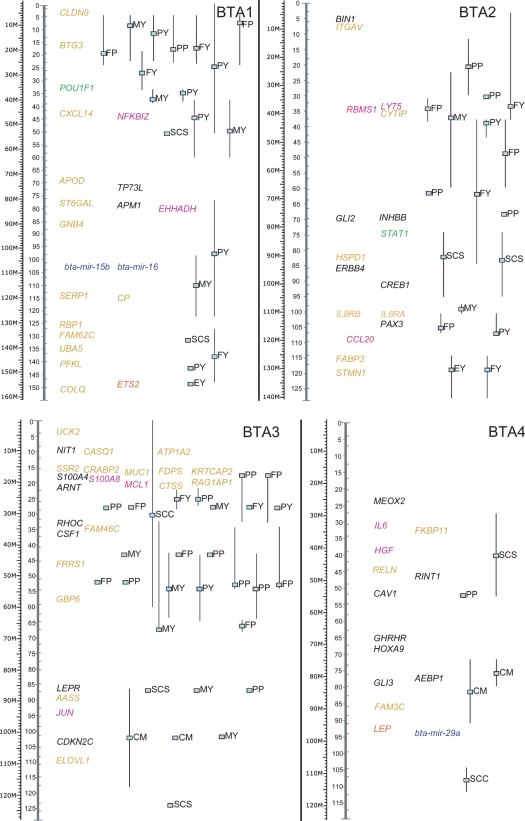

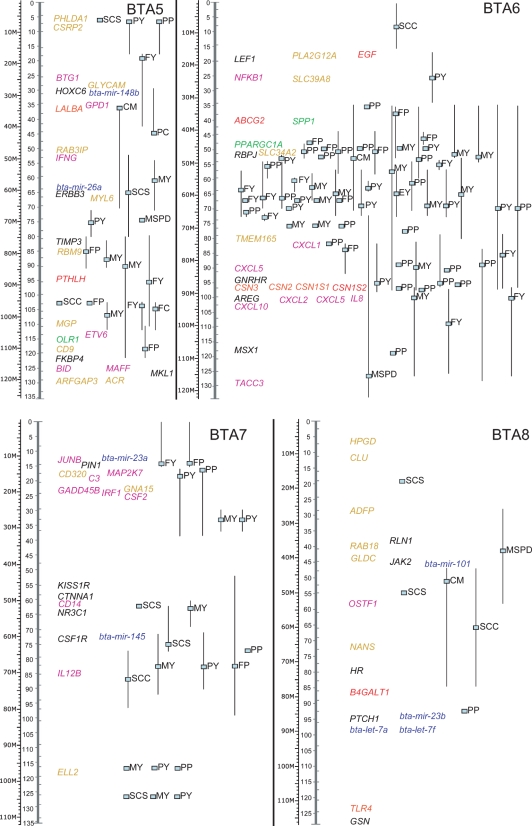

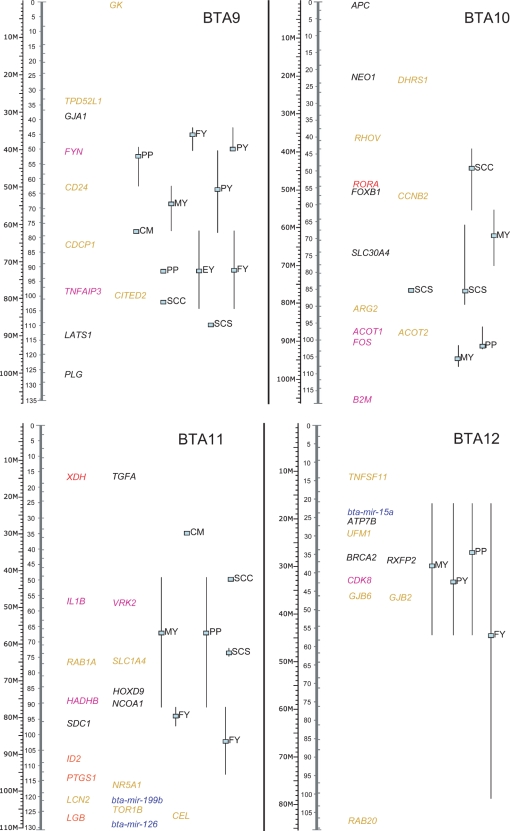

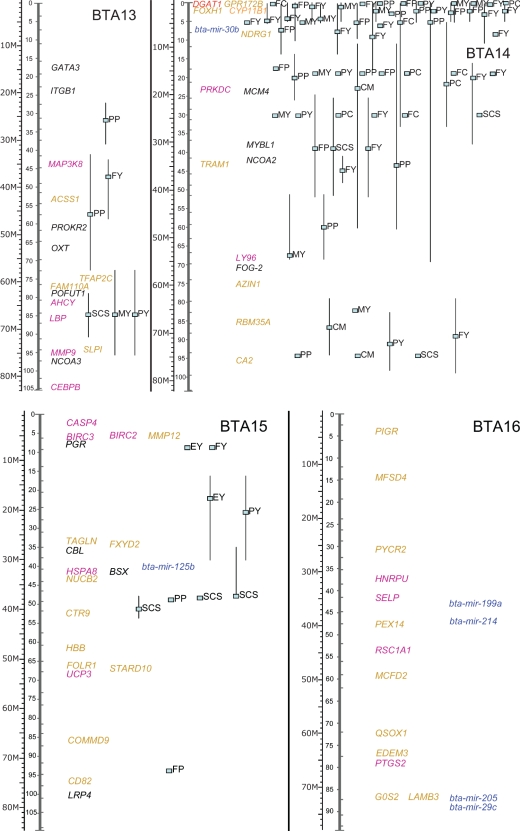

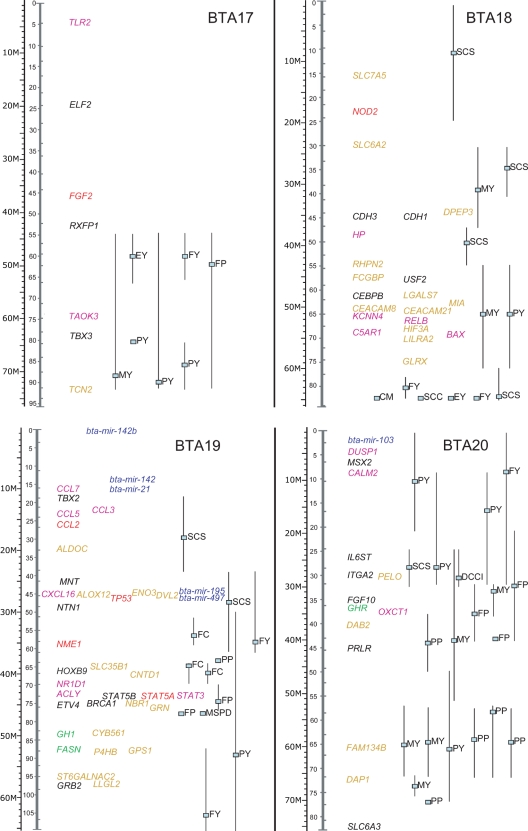

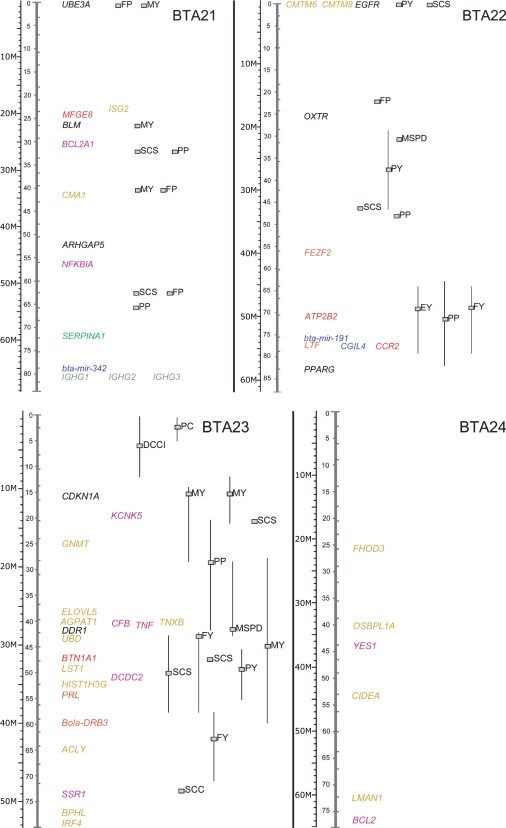

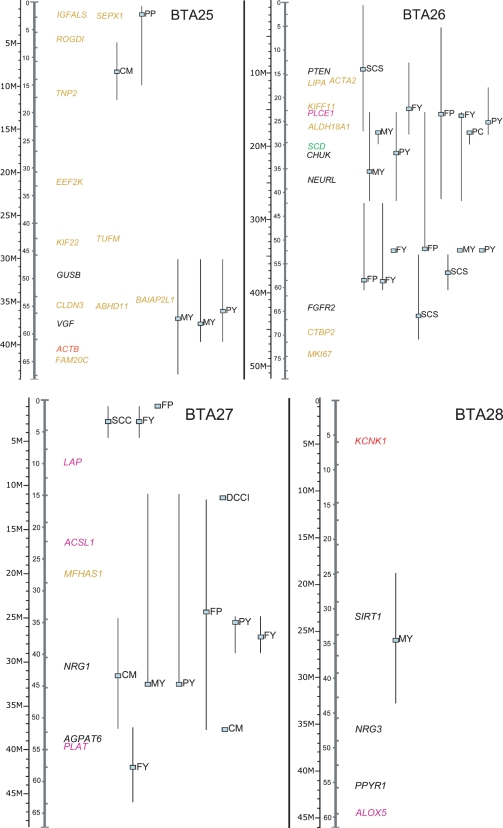

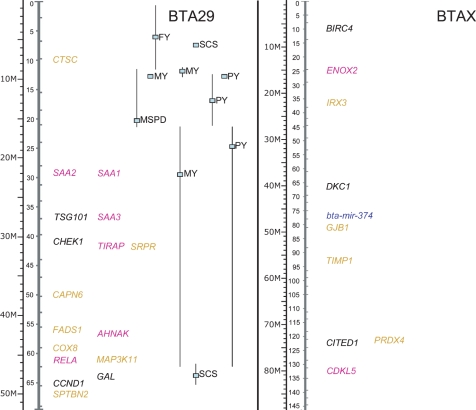
Genetic map of cattle candidate genes and genetic markers for milk production and mastitis. The map includes mouse transgenic and knock-out experiments, QTL for milk and mastitis traits, genes and genetic markers tested for association with milk and mastitis traits, genes with expression patterns associated with milk and mastitis traits, milk protein genes that exist in different genetic variants, miRNAs expressed in mammary gland, and epigenetically regulated gene associated with mammary gland phenotype. The ruler to the extreme left of each figure represents mega-base pairs. The ruler next to the mega-base pairs scale represents distances in centimorgans. Loci are placed at approximate positions on both the sequence and the linkage map. Chromosomes are not drawn to scale. Legend: Transgenics and knock-outs (mouse models). 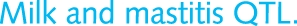

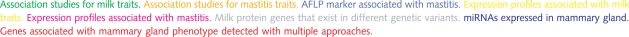

**Figure 2:**
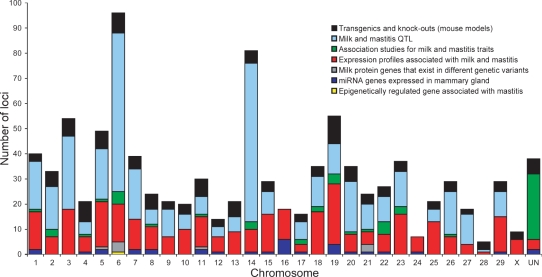
Number of candidate genes and genetic markers for mammary gland development, milk production traits and resistance or susceptibility to mastitis found with different approaches by chromosome.

Genes, QTL, SNPs, AFLP markers and miRNAs representing 934 cattle loci involved in mammary gland development, milk production traits and resistance or susceptibility to mastitis were retrieved from different sources. The results are presented in the form of a genetic marker map (Fig. 1). The collected data include genetic as well as epigenetic background for mammary gland related traits. The database shows putative mammary gland related candidate loci on all chromosomes except on chromosome Y, with the highest number of candidate loci on chromosomes 6, 14 and 19 and the lowest on the chromosomes 28, 24, and X (Fig. 2). The Ingenuity Pathway Analysis identified that among the 44 candidate genes confirmed in multiple studies, 12 loci are involved in inflamatory response and antigen presentation and 10 loci are involved in development and function of connective tissue, muscle development and function as well as development and function of endocrine system. Eight loci are involved in cell mediated immune response and structure and development of lymphoid tissue and the other eight are involved in cellular development, movement and cancer. Three loci were associated with organ morphology, development of reproductive system and amino acid metabolism (Table 2). However, three genes could not be associated with physiological function using Ingenuity Pathway Analysis due to specificies cattle genome (*LGB, BoLA-DRB3* and *CSN1S2*).

**Table 2:** The most promising candidate genes found by matching data sets from independent studies and their *in silico* analysis (level of expression in lactating mouse mammary gland, genetic variability in cattle and their functions).

List of the most promising candidate genes and number of independent studies by approach									*In silico* analysis						
			Association studies		Expression studies					Number of SNPs					
Gene	Gene name	Mouse KOs and transgenic experiments	Milk traits	Mastitis traits	Milk traits^1^	Mastitis traits	Milk protein genetic variants	Epigenetic studies	Expression in lactating mammary gland^2^ (mouse)	Promoter (5 kb upstream)	5′UTR	Exons^3^	Introns (context 100 bp)	3′UTR	Functions^4^
Associated with milk production															
*ABCG2*	*ATP-binding cassette, sub-family G (WHITE), member 2*		+ + +		+				***	0	0	1	1	0	E
*ATP2B2*	*ATPase, Ca++ transporting, plasma membrane 2*	+			+				*	1	0	1	3	0	D
*B4GALT1*	*UDP-Gal:betaGlcNAc beta 1,4- galactosyltransferase, polypeptide 1*	+			+				**	0	0	0	1	0	A
*BTN1A1*	*Butyrophilin, subfamily 1, member A1*	+			+				***	0	0	3 (2)	0	0	C
*CSN1S1*	*Casein alpha s1*		+ + +		+		+	+	***	0	0	1 (1)	0	0	C
*CSN1S2*	*Casein alpha s2*				+		+		NA	0	0	0	0	0	NA
*CSN2*	*Casein beta*	+					+		***	0	0	3 (3)	0	0	B
*CSN3*	*Casein kappa*	+	+ + +		+		+		***	0	0	0	3	0	B
*DGAT1*	*Diacylglycerol O-acyltransferase 1*	+	+ + + + + + +						**	NA	NA	NA	NA	NA	A
*EGF*	*Epidermal growth factor (beta urogastrone)*	+			+				NA	0	0	0	0	0	B
*GHR*	*Growth hormone receptor*		+ + + +						**	0	0	0	0	0	B
*ID2*	*Inhibitor of DNA binding 2, dominant negative helix-loop-helix protein*	+			+				*	0	0	0	1	0	B
*LALBA*	*Lactalbumin, alpha*	+			+		+		***	0	0	0	0	1	A
*LEP*	*Leptin*	+	+ + +		+				**	0	0	8 (3)	4	8	B
*LGB*	*Lactoglobulin, beta*		+ +				+		NA	0	1	1	0	0	NA
*MFGE8*	*Milk fat globule-EGF factor 8 protein*	+			+				***	0	0	0	0	1	A
*NME1*	*Non-metastatic cells 1, protein (NM23A) expressed in*	+			+				NA	0	0	3 (2)	0	1	D
*PRL*	*Prolactin*	+	+ +						NA	0	0	0	0	0	B
*PTHLH*	*Parathyroid hormone-like peptide*	+			+				**	0	0	0	0	0	C
*STAT5A*	*Signal transducer and activator of transcription 5A*	+	+ +						**	0	0	0	0	0	B
*XDH*	*Xanthine dehydrogenase*	+			+				***	0	0	2 (1)	1	0	C
Associated with mastitis resistance															
*ACTB*	*Actin, beta, cytoplasmic*	+				+			*	0	0	0	0	1	D
*C5AR1*	*Complement component 5a receptor 1*					+ +			**	0	0	1	0	0	B
*CD14*	*CD14 antigen*					+ + + +			***	0	1	1	0	0	A
*ETS2*	*E26 avian leukaemia oncogene 2, 3′ domain*	+				+			*	0	0	0	1	1	C
*FEZF2*	*fez family zinc finger 2*			+		+			**	0	0	1 (1)	1	0	E
*IFNG*	*Interferon gamma*					+ + +			**	0	0	0	1	2	D
*IL1B*	*Interleukin 1 beta*					+ + + +			**	0	0	2	0	7	D
*IL6*	*Interleukin 6*					+ +			**	0	0	1	1	1	B
*IL8*	*Interleukin 8*					+ + +			NA	0	0	0	5	7	C
*IL8RA*	*Interleukin 8 receptor, alpha*			+ +					**	0	0	0	0	0	C
*LBP*	*Lipopolysaccharide binding protein*					+ +			***	2	0	5 (2)	0	0	A
*PTGS1*	*Prostaglandin-endoperoxide synthase 1*	+				+			**	0	0	0	0	0	A
*SAA3*	*Serum amyloid A3*					+ +			**	0	0	0	0	0	A
*TLR-2*	*Toll-like receptor 2*					+ +			**	0	0	3 (2)	0	0	A
*TLR-4*	*Toll-like receptor 4*			+ +		+ +			**	0	1	28 (8)	4	2	A
*TNF*	*Tumor necrosis factor*					+ + + +			**	0	0	0	0	0	A
Associated with milk production and mastitis resistance															
*ACLY*	*ATP citrate lyase*				+	+			**	0	0	2	3	0	D
*BoLA-DRB3*	*Major histocompatibility complex, class II, DRB3*		+ + +	+ + +					NA	1	0	5 (5)	0	0	NA
*CCL2*	*Chemokine (C-C motif) ligand 20*		+	+					*	1	0	2 (2)	1	0	C
*KCNK1*	*Potassium channel, subfamily K, member 1*				+	+			***	1	0	3 (1)	1	0	E
*LTF*	*Lactoferrin*		+ +	+	+	+			**	0	2	4	1	0	A
*RORA*	*RAR-related orphan receptor alpha*				+	+			**	0	0	0	0	0	D
*TP53*	*Transformation related protein 53*	+			+	+			*	0	0	1	1	0	D

+, independent study.

1 Candidates suggested by Ron *et al.* (2007) on the basis of three independent expression experiments.

2 Mouse mammary gland expression score: *below median, **median to 10× median, ***above 10× median.

3 Number in brackets represents number of non-synonymous coding SNPs.

4 Top functions: A – inflammatory disease, inflammatory response, antigen presentation, B – connective tissue development and function, skeletal and muscular system development and function, endocrine system development and function, C – cancer, cellular movement and cellular development, D – cell-mediated immune response, lymphoid tissue structure and development, cancer, E – organ morphology, reproductive system development and function and amino acid metabolism.

### Transgenics and knock-outs

Because of its numerous advantages (large amount of mutations, efficient techniques for targeted mutagenesis, precisely described phenotype changes), the mouse model has been used as a tool for identification of phenotype-genotype relationships. The availability of the complete mouse genome sequence allows comparisons with other species and identification of conserved regions (Guenet 2005). Currently, there are 143 genes that, when mutated or expressed as transgenes in mouse, result in phenotypes associated with mammary gland (Table S1).

### Milk and mastitis QTL

There are 344 QTL associated with milk traits in cattle (MY, MSPD, DCCI, PY, PP, EY, FP) and 71 mastitis related traits (CM, SCS and SCC) available in AnimalQTL database. QTL are positioned on all chromosomes except on BTA16, BTA24 and BTAX. The reason why milk and mastitis QTL are spread over such a number of chromosomes might be in the numerous genetic and environmental factors that contribute to animal’s phenotype, including different traits and specific host-pathogen interactions. The highest density of QTL associated with milk traits was found on BTA6 and BTA14 and the highest density of mastitis related QTL on BTA3 and BTA14.

### Association studies

#### SNPs associated with mastitis

Allele-phenotype association studies were performed for milk (MY, milk protein, PP, milk fat and FP) and mastitis (CM and SCS) traits. Association between DNA sequence variation and mammary gland phenotype has been demonstrated for twenty-four candidate genes (Sharif *et al.* 1999; Grisart *et al.* 2002; Blott *et al.* 2003;Kuss *et al.* 2003; Prinzenberg *et al.* 2003; Brym *et al.* 2004, 2005; Cohen-Zinder *et al.* 2005; Khatib *et al.* 2005; Kuss *et al.* 2005; Liefers *et al.* 2005;Leonard *et al.* 2005; Weikard *et al.* 2005; Zhou *et al.* 2005; Cobanoglu *et al.* 2006; do Nascimento *et al.* 2006; He *et al.* 2006; Kaminski *et al.* 2006; Khatib *et al.* 2006; Ron *et al.* 2006; Sanders *et al.* 2006; Kaupe *et al.* 2007; Leyva-Baca *et al.* 2007; Morris *et al.* 2007; Olsen *et al.* 2007; Pant *et al.* 2007; Robitaille *et al.* 2007;Rupp *et al.* 2007; Anton *et al.* 2008; Banos *et al.* 2008; Chebel *et al.* 2008; Hradecka *et al.* 2008; Ganai *et al.* 2009; Huang *et al.* 2008; Kaminski *et al.* 2008; Khatib *et al.* 2008; Macciotta *et al.* 2008; Wang *et al.* 2008). The association between DNA sequence variation and mastitis resistance or susceptibility has been found for ten candidate genes (Sharif *et al.* 1998; Youngerman *et al.* 2004;do Nascimento *et al.* 2006; Sharma *et al.* 2006b; Sugimoto *et al.* 2006; Wojdak-Maksymiec *et al.* 2006; Kaupe *et al.* 2007; Leyva-Baca *et al.* 2007; Pant *et al.* 2007; Rambeaud & Pighetti 2007; Rupp *et al.* 2007; Wang *et al.* 2007; Leyva-Baca *et al.* 2008) (Table S2). The evidence for the association of 11 genes (*ABCG2, BoLA-DRB3, CSN1S1, CSN3, DGAT1, GHR, LGB, LEP, LTF*, *PRL* and *STAT5A*) with mammary gland phenotype and three genes (*IL8RA*, *TLR4* and *BoLA-DRB3*) with mastitis resistance or susceptibility has been reported more than once in different studies.

#### AFLP markers associated with mastitis

Genome screening for QTL is usually costly and highly laborious. Xiao *et al.* (2007) presented a simplified, inexpensive QTL mapping approach by integration of AFLP markers, DNA pooling and bioinformatics tools. Similarly, Sharma *et al.* (2006a) searched for genome-wide QTL-linked AFLP markers for mastitis resistance in Canadian Holsteins. Cows were screened by selective DNA pooling and AFLP technique. Twenty-seven AFLP markers associated with CM were found and the most promising marker named *CGIL4* was then further characterized and mapped to BTA22 q24. However, due to their dominant character, the AFLPs are less informative than SNPs, which have become widely used with the progress of genome sequencing.

### Expression profiles associated with milk production and mastitis

The high throughput technologies such as microarray analysis offer the possibility of studying changes in expression profiles of thousands of genes, in response to infection with a pathogen, simultaneously. Although microarray analysis has become an important tool in animal genomics, there is still the major problem that no clear consensus about the microarray data processing methods for detection of differentially expressed genes exists (Jaffrezic *et al.* 2007). Candidate genes with expression patterns associated with milk production in cattle were identified by Ron *et al.* (2007) by combining their mouse mammary gland gene expression experiments with two other expression experiments (Clarkson *et al.* 2004; Stein *et al.* 2004) using comparative mapping. The results are available as a web tool for candidate genes for QTL (cgQTL database). To date, twelve publications describing 107 genes with expression patterns associated with mastitis cases in cattle using microarrays (Pareek *et al.* 2005; Sugimoto *et al.* 2006; Zheng *et al.* 2006), real-time PCR (Long *et al.* 2001; Lee *et al.* 2003; Pfaffl *et al.* 2003; Schwerin *et al.* 2003; Goldammer *et al.* 2004; Swanson *et al.* 2004) and ELISA (Bannerman *et al.* 2004a,b; Lee *et al.* 2006) have been published (Table S3). The studies were performed in cattle and mouse using pathogens *Streptococcus uberis, Streptococcus agalactiae*, coliforms (i.e. *Escherichia coli*, *Klebsiella pneumoniae*)*, Staphylococcus spp.* (i.e. *aureus*)*, Cornybacterium spp.,* and yeast. Differential expression of eleven genes (*IL6, IL8, CD14, TLR4, IL1B, LBP, TLR2, C5AR1, TNF*, *IFNG* and *SAA3*) during mastitis was confirmed in more than one (two to four) expression experiment, moreover, six genes (*IL6, CD14, TLR4, IL1B, TLR2* and *SAA3*) were found to be differentially expressed in two species (cattle and mouse).

### Milk protein genes

Farrell *et al.* (2004) reported 14 major proteins in bovine milk. Milk protein genes exist in different genetic variants that encode proteins that are slightly different chemically. Numerous investigatiors have focused on the association between certain genetic variants of milk proteins and yield traits, milk composition and technological properties of milk (Buchberger & Dovc 2000). However, the allele-specific effects are very much dependant on genetic background (breed) and experimental model (single locus vs. multi locus effects). Currently there are milk protein variants known for nine milk protein families in bovine milk (Table S4), but only a few of them affect milk traits significantly.

### miRNA genes expressed in mammary gland

miRNAs are a new class of regulatory molecules and could also be involved in the regulation of gene expression in the mammary gland. To date, 32 miRNA genes have been reported to be expressed in the bovine mammary gland (Gu *et al.* 2007). Some of these miRNAs are located in overlapping regions with QTL for milk and/or mastitis traits (Table S5). Recently, several genes have been proven to be regulated *via* miRNAs, but so far none of them in mammary gland-related traits.

For miRNAs, experimentally proven to be expressed in mammary gland, we performed *in silico* searches for target sites and found 359 putative miRNA target sites in candidate genes. Using the Patrocles database, we found polymorphic miRNA target sites for bta-miR-199b, -miR-199a-5p, and -miR-361 in the *IL1B* gene and for –miR-126 in the *CYP11B1* gene. Interestingly, the expression of -miR-199b, -miR-199a-5p and –miR-126 in the bovine mammary gland has already been experimentally confirmed.

### Epigenetic factors

Epigenetic factors have also been demonstrated to be involved in CM (Vanselow *et al.* 2006). Principally, DNA-remethylation around the *STAT5*-binding enhancer in the *CSN1S1* promoter was shown to be associated with shutdown of α_S1_-casein synthesis during acute mastitis. Interestingly, defensin genes *BNBD5* and *LAP* are regulated in an opposite manner to the *CSN1S1* promoter (Vanselow *et al.* 2006), which was also found to be associated with milk traits.

## Discussion

The extensive literature and database search for mammary gland associated candidate genes and genome loci have been performed. We reviewed 934 loci involved in mammary gland development, milk production traits and resistance or susceptibility to mastitis in cattle (Table 1).

**Table 1:** Summary of the data in the database of cattle candidate genes and genetic markers for mammary gland development, milk production traits and resistance or susceptibility to mastitis.

Study approach	Number of loci
Knock-out and transgenic experiments	143
QTL	415
Association studies – milk traits	24
Association studies – mastitis	10
AFLP markers associated with mastitis	27
Expression studies – milk traits	207
Expression studies – mastitis	107
Milk protein genes that exist in different genetic variants	9
miRNAs expressed in mammary gland	32
Epigenetic factors	1
Total	934*

* Unique loci (studies reporting individual gene more than once by different approaches were subtracted from sum).

Our criteria for inclusion of candidate regions into the database were association of the genetic marker with the animal trait, which mainly revealed functional candidates, and sequence similarity revealing structural candidates and map position (overlap with QTL of interest), which allowed identification of positional candidates.

As the data extracted from different sources are often fragmented and controversial, there is an urgent need to integrate information from different sources. Our database consists of cattle candidate loci for mammary gland development, milk production traits and resistance or susceptibility to mastitis comprising 934 loci. The database is available in Excel format and allows searching for loci by name, approach, reference and chromosomal location. The loci in the database are hyperlinked to the relevant public databases (NCBI, MGI, CattleQTLdb, and miRBase). Human and mouse homologs are available for all cattle genes, which represents an important advantage for a comparative approach. In cases when locations for bovine orthologs were unavailable on NCBI’s Map Viewer –*Bos taurus* build (4.0), we defined approximate locations of cattle orthologs by using a bovine-human synteny map. The cattle mammary gland database will serve as a source of candidates for functional studies and development of markers for the new generation of animal breeding tools.

Candidate loci were drawn to the genetic marker map (Fig. 1). The advantage of the map-based review is the identification of overlapping regions populated with candidate loci found by different approaches. A review of the genetic map approach has been previously published for obesity-related loci (Rankinen *et al.* 2006) and was heavily used as an important research tool in obesity studies.

We found 44 candidate genes identified in multiple independent studies using the same or different approaches, of which 22 were associated with milk production, 16 with mastitis and six with both (Table 2). Genes identified with multiple approaches or in multiple analyses using the same approach and/or in regions overlapping with QTL represent promising candidate genes for association with mammary gland development, lactation and resistance or susceptibility to mastitis.

The most promising candidates were further analysed *in silico* (Table 2). A search for expression in lactating mammary gland and polymorphisms (SNPs) in different regions (promoter, 5′UTR, exon, intron and 3′UTR) was performed. To date, there are 159 SNPs reported in 44 of the most promising candidate genes. We found 82 SNPs in exons, 34 in introns (100 bp flanking region), 32 in 3′UTRs, six in promoters and five in 5′UTRs of selected genes. The most polymorphic gene was *TLR4*, with 35 reported SNPs. Genes with a high number of reported polymorphisms were also *LEP* (20 SNPs), *IL8* (12), *IL1B* (9) and *LTF* (7). Additionally, the pathway analysis was used to cluster the genes into five functional networks involved in a variety of biological functions.

Twenty-six genes (*ABCG2*, *ACLY*, *ACTB*, *ATP2B2*, *B4GALT1*, *BoLA-DRB3, BTN1A1*, *CCL2*, *CSN1S2*, *CSN2*, *DGAT1*, *EGF*, *ETS2, FEZF2*, *ID2*, *KCNK1*, *MFGE8*, *NME1*, *LGB, PRL*, *PTGS1*, *PTHLH*, *RORA*, *STAT5A*, *TLR*4 and *XDH*) were found to be associated with mammary gland phenotypes (milk and mastitis traits) using two different study approaches, *LALBA*, *LEP*, *TP53* using three different approaches, and *CSN3*, *CSN1S1* and *LTF* using four different approaches. Twenty-five genes were confirmed in multiple independent studies using the same approach; eleven of them in association studies for milk traits (*ABCG2*, *BoLA-DRB3*, *CSN1S1*, *CSN3*, *DGAT1*, *GHR*, *LEP*, *LGB*, *LTF*, *PRL*, and *STAT5A*), three in association studies for mastitis traits (*BoLA-*DRB3, IL8RA and *TLR4*) and 11 in mastitis expression experiments (*C5AR1, CD14, IFNG, IL1B: IL6, IL8, LBP, SAA3, TLR2, TLR4* and *TNF*). Genes *ABCG2*, *BoLA-DRB3, CSN1S1, CSN3, LEP*, *LTF* and *TLR4* were reported by at least two different approaches and confirmed in at least two independent studies for each approach. None of the miRNA genes overlapped with candidate gene locations, but 10 of them overlapped with QTL regions for different traits (Table S5). Moreover, we performed miRNA target searches in the collected candidate genes and found 359 putative target sites, of which two genes (*IL1B* and *CYP11B*) included polymorphic targets for miRNAs expressed in mammary gland. Those miRNA:mRNA pairs can now be experimentally tested for their possible involvement in the regulation of gene expression in the mammary gland.

The highest density of QTL was found on BTA6 and BTA14. As suggested by Khatkar *et al.* (2004), there are two distinct QTL regions on BTA6 at 49 ± 5.0 cM and 87 ± 7.9 cM. Genes *PPARGC1* (Weikard *et al.* 2005), *SPP1* (Leonard *et al.* 2005) and *ABCG* (Cohen-Zinder *et al.* 2005; Ron *et al.* 2006; Olsen *et al.* 2007), found in association studies for milk traits, are located in proximity of 49 ± 5.0 cM QTL region. In 87 ± 7.9 cM region, which overlaps with several PP QTL, casein genes (*CSN1S1, CSN1S2, CSN2* and *CSN3*) are located. Khatkar *et al.* (2004) detected a genome-wide significant QTL for milk FP and yield close to the centromeric end of BTA14 where the *DGAT1* gene is located. The *DGAT1* gene that overlaps with several milk fat QTL was found in association studies for milk fat (Grisart *et al.* 2002; Kaminski *et al.* 2006; Kaupe *et al.* 2007, Anton *et al.* 2008, Banos *et al.* 2008; Hradecka *et al.* 2008; Kaminski *et al.* 2008) and in murine KO experiments, which resulted in the absence of milk production. As concluded by Grisart *et al.* (2002), the *DGAT1* gene which is involved in triglyceride synthesis is the causative gene affecting milk fat on BTA14. Genes *LEP* (Liefers *et al.* 2005; Banos *et al.* 2008; Chebel *et al.* 2008)*, PRL* (Brym *et al.* 2005; He *et al.* 2006), *CSN3* (Kaminski *et al.* 2006, 2008; Robitaille *et al.* 2007), *DGAT1* (Grisart *et al.* 2002; Kaminski *et al.* 2006; Kaupe *et al.* 2007; Anton *et al.* 2008; Banos *et al.* 2008; Hradecka *et al.* 2008; Kaminski *et al.* 2008) and *STAT5A* (Brym *et al.* 2004; Khatib *et al.* 2008) were reported in association studies for milk traits and in murine KO experiments.

The *LTF* gene on BTA22 was found in mastitis expression experiments (Pfaffl *et al.* 2003), association studies for milk phenotypes (Kaminski *et al.* 2006, 2008) and association studies for mastitis resistance or susceptibility (Wojdak-Maksymiec *et al.* 2006). Lactoferrin (LF), with its strong iron binding properties, is known to have several biological functions including host defence against microbial infection and anti-inflammatory activity. The multifunctional roles of *LTF* were reviewed by Ward *et al.* (2005). The finding that inflammation and involution of the mammary gland induces mammary expression of LF led to the suggestion that the *LTF* gene is a strong functional candidate for mastitis resistance or susceptibility (Kerr & Wellnitz 2003). As reported by Wojdak-Maksymiec *et al.* (2006), two alleles of *LTF*, A and B, were found in the studied population. The highest SCC was found in milk of the AB genotype, whereas the lowest one was found in cows of the AA genotype. The *TLR4* gene on BTA8 was found in mastitis association and expression studies. Its differential expression was confirmed in two different experiments (Goldammer *et al.* 2004; Zheng *et al.* 2006) and association of its sequence polymorphisms with mastitis traits was found in two different studies (Sharma *et al.* 2006b; Wang *et al.* 2007). Therefore, *TLR4* may be a strong candidate for functional studies to enhance mastitis resistance in cattle. The expression of the *FEZF2* gene on BTA22 has been reported to be induced by mastitis and its sequence variation is associated with mastitis resistance or susceptibility (Sugimoto *et al.* 2006); cows susceptible to mastitis have a three-base insertion in a glycine-coding stretch of the gene. Sequence variation of the BTA23-located *BoLA-DRB3* gene has also been reported to be associated with milk traits and mastitis resistance or susceptibility (Sharif *et al.* 1999; do Nascimento *et al.* 2006; Rupp *et al.* 2007). As suggested by do Nascimento *et al.* (2006), this might be due to a direct action of bovine major histocompatibility complex alleles on immune function, whereas effects on production traits might be only indirect and explained by better general health conditions of more productive animals. *PTGS1* (Pfaffl *et al.* 2003)*, ACTB* (Lee *et al.* 2006)*, TP53* (Schwerin *et al.* 2003) and *ETS2* (Zheng *et al.* 2006) genes were found in mastitis expression studies and murine KO experiments that resulted in increased tumorigenesis of mammary gland and abnormal lactation. To address the developmental-specific expression profiles in the mammary gland, a new specialized microarray containing about 6000 highly enriched unique sequences from mouse mammary libraries (mammochip) has been developed and applied for expression profiling of the mouse mammary gland during development (Miyoshi *et al.* 2002). Comparison of gene expression in the wild type lactating and virgin mammary gland and in KO for the *inhibitor of differentiation 2* (*Id2*) gene revealed four distinct groups of genes showing different expression profiles.

Major lactoprotein genes (*CSN1S1, CSN1S2, CSN2, CSN3, LALBA* and *LGB*) exist in different genetic variants that code for chemically different protein variants. The genetic variants of milk proteins have diverse effects on milk composition and cheese making ability. It is possible that effects on milk composition and cheese making ability are not the direct consequence of polymorphisms at lactoprotein gene loci but rather the consequence of QTL linked to the different genetic variants of these genes. Besides protein variant studies, milk protein genes have been also identified by other study approaches: (i) *CSN1S1* in an epigenetic study (Vanselow *et al.* 2006), expression experiments for milk traits (Ron *et al.* 2007), and in association studies for milk traits (Prinzenberg *et al.* 2003; Kuss *et al.* 2005; Sanders *et al.* 2006), (ii) *CSN2* in knock-out experiments in mice which resulted in abnormal lactation and abnormal milk composition, (iii) *CSN3* gene in association studies for milk traits (Kaminski *et al.* 2006; Robitaille *et al.* 2007), expression experiments for milk traits (Ron *et al.* 2007) and in KO experiments in mice which resulted in abnormal lactation and abnormal milk composition, (iv) *LALBA* in KO experiments in mice which resulted in abnormal mammary gland morphology and in abnormal milk composition, expression experiments for milk traits (Ron *et al.* 2007) and (v) a *LGB* in association study for milk traits (Kuss *et al.* 2003).

Epigenetic modifications to the DNA sequence and associated chromatin are also known to regulate gene expression and contribute significantly to the phenotype. Variation in the epigenotype between genetically identical individuals can be associated with phenotypic differences. Moreover, the recent evidence suggests that the epigenome can be affected by environmental factors and that these changes can last a lifetime (Whitelaw & Whitelaw 2006). The *CSN1S1* gene has been reported to be epigenetically regulated during mastitis (Vanselow *et al.* 2006).

Some of the genes which we identified on the cross-cut between different approaches or which were reported in multiple independent studies using the same approach were already identified and verified to affect QTL in cattle (i.e. *ABCG2* and *DGAT1*), while others represent background for subsequent functional studies. Possible criterion to determine priority for further candidate gene analysis can be differential expression of the gene in the target organ, known physiological role to the trait (Ron *et al.* 2007) and positional overlapping with QTL of interest. The current database of cattle candidate genes and genetic markers for mammary gland development, milk production traits and resistance or susceptibility to mastitis consists of 934 unique loci. The project is ongoing and we plan to update the database periodically with further publications.

## Supporting information

**Table S1** Transgenic and knockout murine models related to mammary gland phenotypes.

**Table S2** Genes tested for association with milk and mastitis traits.

**Table S3** Genes with expression patterns associated with mastitis.

**Table S4** Milk protein genes and their variants (adapted from Farrell *et al.* 2004 and Buchberger & Dovc 2000).

**Table S5** miRNAs expressed in mammary gland (adapted from Gu *et al.* 2007).

As a service to our authors and readers, this journal provides supporting information supplied by the authors. Such materials are peer-reviewed and may be re-organized for online delivery, but are not copy-edited or typeset. Technical support issues arising from supporting information (other than missing files) should be addressed to the authors.
