# Supplementary material for: Database of cattle candidate genes and genetic markers for milk production and mastitis
Source: Anim Genet. 2009 Dec;40(6):832–51. doi: 10.1111/j.1365-2052.2009.01921.x (PMC2779988; doi:10.1111/j.1365-2052.2009.01921.x)
Supplement: Supplementary file 1 [file age0040-0832-SD1.pdf]

Table S1 Transgenic and knock-out murine models related to mammary gland phenotypes.

Mouse gene		Human ortholog		Cattle ortholog			Gene name	Phenotype ontology terms
Gene	Chr.	Gene	Chr.	Gene	Chr.	Location (bp)		
Adipoq	16	*ADIPOQ*	3 (q27)	*APM1*	1	82 M	*adiponectin, C1Q and collagen domain containing*	abnormal lactation
Ets2	16	*ETS2*	21 (q22.2)	*ETS2*	1	154.594.100-154.622.300	*E26 avian leukemia oncogene 2, 3' domain*	mammary gland hyperplasia, mammary gland tumor
Trp63	16	*TP63*	3 (q28)	*TP73L*	1	75 M	*transformation related protein 63*	absent mammary gland
Bin1	18	*BIN1*	2 (q14)	*BIN1*	2	5.553.000-5.696.000	*bridging integrator 1*	mammary gland tumor
Erbb4	1	*ERBB4*	2 (q33.3-q34)	*ERBB4*	2	94 M	*v-erb-a erythroblastic leukemia viral oncogene homolog 4 (avian)*	abnormal mammary gland morphology, abnormal mammary gland growth during lactation, abnormal mammary gland growth during pregnancy, abnormal lactation
Gli2	1	*GLI2*	2 (q14)	*GLI2*	2	75.860.000-76.480.000	*GLI-Kruppel family member GLI2*	abnormal mammary gland development
Pax3	1	*PAX3*	2 (q35)	*PAX3*	2	114.961.000-115.156.000	*paired box gene 3*	abnormal mammary gland embryonic development
Inhbb	1	*INHBB*	2 (cen-q13)	*INHBB*	2	75.596.700-75.603.800	*inhibin beta*	abnormal mammary gland morphology, abnormal lactation, abnormal milk ejection
Creb1	1	*CREB1*	2 (q34)	*CREB1*	2	88 M	*cAMP responsive element binding protein 1*	lactation
Arnt	3	*ARNT*	1 (q21)	*ARNT*	3	21.424.000-21.513.000	*aryl hydrocarbon receptor nuclear translocator*	abnormal mammary gland growth during pregnancy, abnormal lactation
Cdkn2c	4	*CDKN2C*	1 (p32)	*CDKN2C*	3	102.127.000-102.138.100	*cyclin-dependent kinase inhibitor 2C (p18, inhibits CDK4)*	mammary gland hyperplasia
Csf1	3	*CSF1*	1 (p21-p13)	*CSF1*	3	36.161.600-36.196.300	*colony stimulating factor 1 (macrophage)*	abnormal branching of the mammary ductal tree, abnormal mammary gland growth during pregnancy, abnormal lactation
Lepr	4	*LEPR*	1 (p31)	*LEPR*	3	85.560.000-85.748.000	*leptin receptor*	abnormal lactation
Nit1	1	*NIT1*	1 (q21-q22)	*NIT1*	3	9.299.900-9.307.100	*nitrilase 1*	abnormal branching of the mammary ductal tree
Rhoc	3	*RHOC*	1 (p13.1)	*RHOC*	3	33.137.200-33.152.900	*ras homolog gene family, member C*	mammary gland tumor, mammary adenocarcinoma

S100a4	3	*S100A4*	1 (q21)	*S100A4*	3	18.220.700-18.226.400	*S100 calcium binding protein A4*	mammary gland tumor, mammary adenocarcinoma
Cav1	6	*CAV1*	7 (q31.1)	*CAV1*	4	53.980.000-54.069.000	*caveolin 1, caveolae protein*	lactation
Ghrhr	6	*GHRHR*	7 (p14)	*GHRHR*	4	67.998.000-68.038.000	*growth hormone releasing hormone receptor*	abnormal lactation
Gli3	13	*GLI3*	7 (p13)	*GLI3*	4	81.580.000-82.110.000	*GLI-Kruppel family member GLI3*	abnormal mammary gland embryonic development
Hoxa9	6	*HOXA9*	7 (p15-p14)	*HOXA9*	4	71.607.400-71.615.300	*homeo box A9*	abnormal branching of the mammary ductal tree, abnormal lactation, mammary gland hypoplasia
Lep	6	*LEP*	7 (q31.3)	*LEP*	4	95.665.000-95.707.000	*leptin*	abnormal branching of the mammary ductal tree
Meox2	12	*MEOX2*	7 (p22.1-p21.3)	*MEOX2*	4	25.002.000-25.192.000	*mesenchyme homeobox 2*	abnormal mammary gland development, mammary gland hyperplasia, mammary gland tumor
Aebp	11	*AEBP*	7 (p13)	*AEBP1*	4	79.944.600-79.959.800	*AE binding protein 1*	abnormal involution of the mammary gland
Rint1	5	*RINT1*	7 (q22.2)	*RINT1*	4	48,564,200-48,576,600	*RAD50 interactor 1*	mammary gland tumor, mammary adenocarcinoma
Erbb3	10	*ERBB3*	12 (q13)	*ERBB3*	5	61.765.000-61.817.000	*v-erb-b2 erythroblastic leukemia viral oncogene homolog 3 (avian)*	abnormal branching of the mammary ductal tree
Pthlh	6	*PTHLH*	12 (p12.1-p11.2)	*PTHLH*	5	88.027.900-88.058.000	*parathyroid hormone-like peptide*	lactation
Fkbp4	6	*FKBP4*	12 (p13.33)	*FKBP4*	5	113.810.400-113.829.900	*FK506 binding protein 4*	abnormal mammary gland development
Hoxc6	15	*HOXC6*	12 (q13.3)	*HOXC6*	5	28.831.000-28.907.000	*homeo box C6*	abnormal mammary gland physiology, abnormal lactation
Lalba	15	*LALBA*	12 (q13)	*LALBA*	5	34.385.300-34.390.400	*lactalbumin, alpha*	abnormal mammary gland morphology, abnormal milk composition, abnormal lactation
Timp3	10	*TIMP3*	22 (q12.3)	*TIMP3*	5	76.612.000-76.756.000	*tissue inhibitor of metalloproteinase 3*	abnormal involution of the mammary gland, abnormal lactation

Mkl1	15	*MKL1*	22 (q13)	*MKL1*	5	118.808.000-119.086.000	*MKL (megakaryoblastic leukemia)/myocardin-like 1*	abnormal involution of the mammary gland, abnormal mammary gland growth during lactation, abnormal lactation, abnormal milk ejection
Areg	5	*AREG*	4 (q13-q21)	*AREG*	6	92.446.100-92.476.500	*amphiregulin*	abnormal branching of the mammary ductal tree, abnormal mammary gland growth during pregnancy, abnormal lactation
Csn2	5	*CSN2*	4 (q21.1)	*CSN2*	6	88.322.000-88.345.400	*casein beta*	abnormal milk composition, abnormal lactation
Csn3	5	*CSN3*	4 (q21.1)	*CSN3*	6	88.511.600-88.544.300	*casein kappa*	abnormal milk composition, abnormal lactation
Egf	3	*EGF*	4 (q25)	*EGF*	6	16.740.000-16.998.000	*epidermal growth factor (beta urogastrone)*	abnormal branching of the mammary ductal tree, abnormal mammary gland growth during pregnancy, abnormal lactation
Lef1	3	*LEF1*	4 (q23-q25)	*LEF1*	6	18.396.000-18.689.000	*lymphoid enhancer binding factor 1*	absent mammary gland
Gnrhr	6	*GNRHR*	4 (q21.2)	*GNRHR*	6	86.093.000-86.137.000	*growth hormone releasing hormone receptor*	lactation
Msx1	5	*MSX1*	4 (p16.3-p16.1)	*MSX1*	6	108.681.500-108.692.200	*homeo box, msh-like 1*	abnormal mammary gland development
Rbpj	5	*RBPJ*	4 (p15.2)	*RBPJ*	6	47.015.000-47.292.000	*recombination signal binding protein for immunoglobulin kappa J region*	abnormal mammary gland growth during pregnancy
Csf1r	18	*CSF1R*	5 (q33-q35)	*CSF1R*	7	60.825.000-60.904.000	*colony stimulating factor 1 receptor*	abnormal mammary gland during pregnancy
Kiss1r	10	*KISS1R*	19 (p13.3)	*KISS1R*	7	46 M	*KISS1 receptor*	mammary gland hypoplasia
Nr3c1	18	*NR3C1*	5 (q31.3)	*NR3C1*	7	53.876.000-54.166.000	*nuclear receptor subfamily 3, group C, member 1*	abnormal mammary gland morphology, abnormal mammary gland growth during pregnancy
Pin1	9	*PIN1*	19 (p13)	*PIN1*	7	12.875.500-12.906.100	*protein (peptidyl-prolyl cis/trans isomerase) NIMA-interacting 1*	abnormal mammary gland growth during pregnancy
Ctnna1	18	*CTNNA1*	5 (q31)	*CTNNA1*	7	49.180.000-49.740.000	*catenin (cadherin associated protein), alpha 1*	abnormal mammary gland morphology, abnormal lactation, abnormal milk composition
B4galt1	4	*B4GALT1*	9 (p13)	*B4GALT1*	8	78.943.000-79.076.000	*UDP-Gal:betaGlcNAc beta 1,4-galactosyltransferase, polypeptide*	abnormal lactation, abnormal milk composition

							1	
Hr	14	*HR*	8 (p21.2)	*HR*	8	72.484.000-72.523.000	*hairless*	abnormal mammary gland morphology
Jak2	19	*JAK2*	9 (p24)	*JAK2*	8	41.497.000-41.664.000	*Janus kinase 2*	abnormal mammary gland growth during lactation, abnormal mammary gland growth during pregnancy, abnormal lactation
Ptch1	13	*PTCH1*	9 (q22.3)	*PTCH1*	8	86.499.000-86.673.000	*patched homolog 1*	abnormal mammary gland development
Rln1	19	*RLN1*	9 (p24.1)	*RLN1*	8	35 M	*relaxin 1*	abnormal mammary gland development,
Gsn	2	*GSN*	9 (q33)	*GSN*	8	116.083.000-116.152.000	*gelsolin*	abnormal branching of the mammary ductal tree, abnormal mammary gland growth during pregnancy
Gja1	10	*GJA1*	6 (q21-q23.2)	*GJA1*	9	31.049.200-31.081.700	*gap junction membrane channel protein alpha 1*	abnormal mammary gland morphology, abnormal mammary gland growth during pregnancy, abnormal milk ejection, abnormal lactation
Lats1	10	*LATS1*	6 (q24-q25.1)	*LATS1*	9	90.004.000-90.102.000	*large tumor suppressor*	abnormal mammary gland development
Plg	17	*PLG*	6 (q26)	*PLG*	9	100.391.000-100.510.000	*plasminogen*	abnormal mammary gland morphology, abnormal involution of the mammary gland, abnormal mammary gland growth during lactation, abnormal lactation, mammary gland tumor, mammary adenocarcinoma
Apc	18	*APC*	5 (q21-q22)	*APC*	10	567.000-807.000	*adenomatosis polyposis coli*	mammary gland tumor, mammary adenocarcinoma
Foxb1	9	*FOXB1*	15 (q21-q26)	*FOXB1*	10	50.628.580-50.631.250	*forkhead box B1*	abnormal milk ejection, abnormal lactation
Neo1	9	*NEO1*	15 (q22.3-q23)	*NEO1*	10	20.239.000-20.478.000	*neogenin*	abnormal mammary gland development
Slc30a4	2	*SLC30A4*	15 (q11.2-q21.3)	*SLC30A4*	10	66.548.000-66.612.000	*solute carrier family 30 (zinc transporter), member 4*	abnormal milk composition, abnormal lactation
Hoxd9	2	*HOXD9*	2 (q31.1)	*HOXD9*	11	72 M	*homeo box D9*	mammary gland hypoplasia
Id2	12	*ID2*	2 (p25)	*ID2*	11	91.373.900-91.379.700	*inhibitor of DNA binding 2*	abnormal mammary gland growth during pregnancy
Ncoa1	12	*NCOA1*	2 (p23)	*NCOA1*	11	76.470.000-	*nuclear receptor coactivator 1*	abnormal mammary gland development

						76.880.000		
Ptgs1	2	*PTGS1*	9 (q32-q33.3)	*PTGS1*	11	96.337.000-96.402.000	*prostaglandin-endoperoxide synthase 1*	abnormal lactation
Sdc1	12	*SDC1*	2 (p24.1)	*SDC1*	11	80.965.000-81.025.000	*syndecan 1*	mammary gland hyperplasia
Tgfa	6	*TGFA*	2 (p13)	*TGFA*	11	14.415.000-14.702.000	*transforming growth factor alpha*	abnormal branching of the mammary ductal tree, abnormal mammary gland growth during pregnancy, abnormal lactation
Xdh	17	*XDH*	2 (p23.1)	*XDH*	11	14.760.000-14.910.000	*xanthine dehydrogenase*	abnormal involution of the mammary gland, abnormal mammary gland growth during lactation, abnormal milk composition, lactation, abnormal lactation
Atp7b	8	*ATP7B*	13 (q14.3)	*ATP7B*	12	20.773.000-20.893.000	*ATPase, Cu++ transporting, beta polypeptide*	abnormal mammary gland physiology, lactation
Brca2	5	*BRCA2*	13 (q12.3)	*BRCA2*	12	28.376.000-28.506.000	*breast cancer 2*	mammary adenocarcinoma
Rxfp2	5	*RXFP2*	13 (q13.1)	*RXFP2*	12	28.967.000-29.117.000	*relaxin/insulin-like family peptide receptor 2*	abnormal nipple morphology, abnormal lactation
Gata3	2	*GATA3*	10 (p15)	*GATA3*	13	14,680,700-14,689,200	*GATA binding protein 3*	abnormal mammary gland morphology, abnormal nipple morphology
Ncoa3	2	*NCOA3*	20 (q12)	*NCOA3*	13	77.018.000-77.091.000	*nuclear receptor coactivator 3*	abnormal branching of the mammary ductal tree
Oxt	2	*OXT*	20 (p13)	*OXT*	13	52.672.300-52.674.540	*oxytocin*	abnormal mammary gland growth during lactation, abnormal milk ejection, abnormal lactation
Pofut	2	*POFUT1*	20 (q11)	*POFUT1*	13	62.525.000-62.625.000	*protein O-fucosyltransferase 1*	abnormal mammary gland growth during pregnancy
Prokr2	2	*PROKR2*	20 (p12.3)	*PROKR2*	13	47.730.000-47.748.300	*prokineticin receptor 2*	abnormal mammary gland morphology
Itgb1	8	*ITGB1*	10 (p11.2)	*ITGB1*	13	19.205.000-19.310.000	*integrin beta 1*	abnormal mammary gland morphology, abnormal mammary gland growth during lactation, resistance to mammary neoplasm
Dgat1	15	*DGAT1*	8 (q24.3)	*DGAT1*	14	442.100-448.800	*diacylglycerol O-acyltransferase 1*	abnormal lactation
Mcm4	16	*MCM4*	8 (q11.2)	*MCM4*	14	19.201.500-19.231.400	*minichromosome maintenance deficient 4 homolog (S, cerevisiae)*	mammary gland tumor, mammary adenocarcinoma
Mybl1	1	*MYBL1*	8 (q22)	*MYBL1*	14	30.694.000-30.779.000	*myeloblastosis oncogene-like 1*	abnormal lactation

Ncoa2	1	*NCOA2*	8 (q13.3)	*NCOA2*	14	33,980,000-34,420,000	*nuclear receptor coactivator 2*	abnormal branching of the mammary ductal tree
Zfpm2	15	*ZFPM2*	8 (q23)	*FOG-2*	14	56.360.000-57.730.000	*zinc finger protein, multitype 2*	abnormal mammary gland morphology, abnormal involution of the mammary gland
Cbl	9	*CBL*	11 (q23.3)	*CBL*	15	28.289.000-28.449.000	*Casitas B-lineage lymphoma*	abnormal branching of the mammary ductal tree
Pgr	9	*PGR*	11 (q22-q23)	*PGR*	15	6.319.000-6.610.000	*progesterone receptor*	abnormal branching of the mammary ductal tree
Bsx	9	*BSX*	11 (q24.1)	*BSX*	15	32.265.200-32.274.400	*brain specific homeobox*	abnormal involution of the mammary gland, abnormal mammary gland growth during lactation
Lrp4	2	*LRP4*	11 (p11.2-p12)	*LRP4*	15	77.111.000-77.205.000	*low density lipoprotein receptor-related protein 4*	abnormal mammary gland development, abnormal mammary gland embryonic development
Elf5	2	*ELF2*	11 (p13-p12)	*ELF2*	17	19.803.000-20.051.000	*E74-like factor 5*	abnormal lactation
Rxfp1	3	*RXFP1*	4 (q32.1)	*RXFP1*	17	42.244.000-42.563.000	*relaxin/insulin-like family peptide receptor 1*	abnormal nipple morphology, abnormal lactation
Tbx3	5	*TBX3*	12 (q24.1)	*TBX3*	17	63.292.300-63.314.900	*T-box 3*	abnormal mammary gland embryonic development, absent mammary gland, mammary gland hypoplasia
Cdh1	8	*CDH1*	16 (q22.1)	*CDH1*	18	35.014.000-35.191.000	*cadherin 1*	abnormal mammary gland morphology, abnormal mammary gland growth during lactation, abnormal mammary gland growth during pregnancy, abnormal milk composition, abnormal lactation, mammary adenocarcinoma, mammary gland tumor
Cdh3	8	*CDH3*	16 (q22.1)	*CDH3*	18	34.956.000-35.070.000	*cadherin 3*	mammary alveolar hyperplasia, mammary ductal hyperplasia
Usf2	7	*USF2*	19 (q13)	*USF2*	18	45.400.000-45.423.500	*upstream transcription factor 2*	lactation
Cebpb	2	*CEBPB*	20 (q13.1)	*CEBPB*	18	48 M	*CCAAT/enhancer binding protein (C/EBP), beta*	abnormal mammary gland morphology, abnormal branching of the mammary ductal tree, abnormal mammary gland physiology, abnormal lactation
Brca1	11	*BRCA1*	17 (q21)	*BRCA1*	19	44.428.000-	*breast cancer 1*	abnormal mammary gland morphology,

						44.600.000		abnormal involution of the mammary gland, abnormal branching of the mammary ductal tree, abnormal mammary gland growth during pregnancy, mammary ductal hyperplasia, mammary gland hyperplasia, mammary adenocarcinoma, mammary gland tumor
Etv4	11	*ETV4*	17 (q21)	*ETV4*	19	44.849.000-44.887.000	*ets variant gene 4 (E1A enhancer binding protein, E1AF)*	abnormal branching of the mammary ductal tree, mammary gland tumor
Grb2	11	*GRB2*	17 (q24-q25)	*GRB2*	19	57.741.000-58.069.000	*growth factor receptor bound protein 2*	mammary gland tumor, mammary adenocarcinoma
Hoxb9	11	*HOXB9*	17 (q21.3)	*HOXB9*	19	39.069.900-39.079.700	*homeo box B9*	mammary gland hypoplasia
Mnt	11	*MNT*	17 (p13.3)	*MNT*	19	25 M	*max binding protein*	abnormal mammary gland morphology, abnormal involution of the mammary gland, mammary gland tumor, mammary adenocarcinoma
Nme1	11	*NME1*	17 (q21.3)	*NME1*	19	36.604.000-36.670.000	*expressed in non-metastatic cells 1, protein*	abnormal branching of the mammary ductal tree, abnormal lactation, lactation
Ntn1	11	*NTN1*	17 (p13-p12)	*NTN1*	19	28.860.000-29.350.000	*netrin 1*	abnormal mammary gland development
Stat5a	11	*STAT5A*	17 (q11.2)	*STAT5A*	19	43.713.000-43.764.000	*signal transducer and activator of transcription 5A*	abnormal mammary gland morphology, abnormal involution of the mammary gland, abnormal branching of the mammary ductal tree, abnormal mammary gland growth during lactation, abnormal mammary gland growth during pregnancy, abnormal mammary gland physiology, abnormal milk ejection, lactation, abnormal lactation
Stat5b	11	*STAT5B*	17 (q11.2)	*STAT5B*	19	43.628.000-43.719.000	*signal transducer and activator of transcription 5B*	abnormal mammary gland physiology, lactation, abnormal lactation
Tbx2	11	*TBX2*	17 (q23)	*TBX2*	19	10.933.700-10.957.300	*T-box 2*	abnormal mammary gland development
Trp53	11	*TP53*	17 (p13.1)	*TP53*	19	27.870.400-27.901.300	*transformation related protein 53*	mammary gland tumor, mammary adenocarcinoma
Fgf10	13	*FGF10*	5 (p13-	*FGF10*	20	32.330.000-	*fibroblast growth factor 10*	abnormal branching of the mammary ductal

			p12)			32.576.000		tree, abnormal mammary gland embryonic development
Il6st	13	*IL6ST*	5 (q11)	*IL6ST*	20	24.760.000-24.891.000	*interleukin 6 signal transducer*	abnormal involution of the mammary gland
Itga2	13	*ITGA2*	5 (q23-q31)	*ITGA2*	20	27.620.000-27.892.000	*integrin alpha 2*	abnormal branching of the mammary ductal tree
Msx2	13	*MSX2*	5 (q34-q35)	*MSX2*	20	6.600.500-6.612.800	*homeo box, msh-like 2*	abnormal mammary gland embryonic development
Prlr	15	*PRLR*	5 (p14-p13)	*PRLR*	20	41.385.000-41.541.000	*prolactin receptor*	abnormal lactation, lactation
Slc6a3	13	*SLC6A3*	5 (p15.3)	*SLC6A3*	20	75.011.000-75.087.000	*solute carrier family 6 (neurotransmitter transporter, dopamine), member 3*	abnormal lactation, lactation
Arhgap5	12	*ARHGAP5*	14 (q12)	*ARHGAP5*	21	43.440.800-43.450.500	*Rho GTPase activating protein 5*	abnormal mammary gland development
Blm	7	*BLM*	15 (q26.1)	*BLM*	21	21.536.000-21.705.000	*Bloom syndrome homolog (human)*	mammary adenocarcinoma, mammary gland tumor
Mfge8	7	*MFGE8*	15 (q25)	*MFGE8*	21	20.226.000-20.264.000	*milk fat globule-EGF factor 8 protein*	abnormal mammary gland morphology, abnormal involution of the mammary gland, abnormal mammary gland growth during lactation, abnormal milk composition, abnormal lactation
Ube3a	7	*UBE3A*	15 (q11-q13)	*UBE3A*	21	229.000-388.000	*ubiquitin protein ligase E3A*	abnormal mammary gland growth during pregnancy
Egfr	11	*EGFR*	7 (p12)	*EGFR*	22	480.000-920.000	*epidermal growth factor receptor*	abnormal mammary gland morphology, abnormal lactation
Oxtr	6	*OXTR*	3 (p25)	*OXTR*	22	18.278.400-18.302.600	*oxytocin receptor*	abnormal lactation
Pparg	6	*PPARG*	3 (p25)	*PPARG*	22	58.253.000-58.435.000	*peroxisome proliferative activated receptor, gamma*	abnormal mammary gland development, abnormal mammary gland growth durin pregnancy
Atp2b2	6	*ATP2B2*	3 (p25.3)	*ATP2B2*	22	55.620.000-56.070.000	*ATPase, Ca++ transporting, plasma membrane 2*	lactation
Btn1a1	13	*BTN1A1*	6 (p22.1)	*BTN1A1*	23	31.593.400-31.609.100	*butyrophilin, subfamily 1, member A1*	abnormal mammary gland morphology, abnormal milk composition, abnormal lactation

Ddr1	17	*DDR1*	6 (p21.3)	*DDR1*	23	28.085.300-28.119.800	*discoidin domain receptor family, member 1*	abnormal mammary gland morphology, abnormal lactation
Prl	13	*PRL*	6 (p22.2-p21.3)	*PRL*	23	35.566.300-35.587.800	*prolactin*	abnormal mammary gland morphology, abnormal branching of the mammary ductal tree, lactation
Cdkn1a	17	*CDKN1A*	6 (p21.2)	*CDKN1A*	23	10.900.300-10.921.000	*cyclin-dependent kinase inhibitor 1A (P21)*	abnormal mammary gland growth during lactation, mammary gland tumor, mammary adenocarcinoma
Actb	5	*ACTB*	7 (p15-p12)	*ACTB*	25	40.632.600-40.641.100	*actin, beta, cytoplasmic*	mammary gland tumor, mammary adenocarcinoma,
Gusb	5	*GUSB*	7 (q21.11)	*GUSB*	25	32 M	*glucuronidase, beta*	abnormal lactation
Vgf	5	*VGF*	7 (q22)	*VGF*	25	37.705.200-37.710.900	*VGF nerve growth factor inducible*	abnormal lactation
Chuk	19	*CHUK*	10 (q24-q25)	*CHUK*	26	21.354.000-21.460.000	*conserved helix-loop-helix ubiquitous kinase*	abnormal lactation
Fgfr2	7	*FGFR2*	10 (q26)	*FGFR2*	26	41.984.000-42.251.000	*fibroblast growth factor receptor 2*	abnormal mammary gland embryonic development, absent mammary gland
Neurl	19	*NEURL*	10 (q25.1)	*NEURL*	26	24.531.000-24.712.000	*neuralized-like homolog (Drosophila)*	abnormal lactation, lactation
Pten	19	*PTEN*	10 (q23.3)	*PTEN*	26	9.806.000-10.043.000	*phosphatase and tensin homolog*	mammary gland tumor, mammary adenocarcinoma
Agpat6	8	*AGPAT6*	8 (p11.21)	*AGPAT6*	27	38.923.000-38.965.000	*1-acylglycerol-3-phosphate O-acyltransferase 6 (lysophosphatidic acid acyltransferase, zeta)*	abnormal mammary gland morphology, abnormal milk composition, lactation, abnormal lactation
Nrg1	8	*NRG1*	8 (p21-p12)	*NRG1*	27	30.350.000-30.890.000	*neuregulin 1*	abnormal mammary gland growth during pregnancy
Nrg3	14	*NRG3*	10 (q22-q23)	*NRG3*	28	35.510.000-38.650.000	*neuregulin 3*	abnormal mammary gland development
Ppyr1	14	*PPYR1*	10 (q11.2)	*PPYR1*	28	41.790.200-41.794.500	*pancreatic polypeptide receptor 1*	abnormal mammary gland growth during pregnancy
Sirt1	10	*SIRT1*	10 (q21.3)	*SIRT1*	28	23.593.000-23.660.000	*sirtuin 1*	abnormal mammary gland development, abnormal branching of the mammary ductal tree, abnormal mammary gland growth during pregnancy, abnormal mammary gland morphology, abnormal lactation

Tsq101	7	*TSG101*	11 (p15)	*TSG101*	29	27.436.000-27.540.000	*tumor susceptibility gene 101*	abnormal lactation
Ccnd1	7	*CCND1*	11 (q13)	*CCND1*	29	48.733.800-48.757.500	*cyclin D1*	abnormal branching of the mammary ductal tree, abnormal mammary gland growth during lactation, abnormal mammary gland growth during pregnancy, abnormal lactation, resistance to mammary neoplasm
Chek1	9	*CHEK1*	11 (q24-q24)	*CHEK1*	29	30.568.000-30.613.000	*checkpoint kinase 1 homolog (S, pombe)*	abnormal mammary gland growth during pregnancy, abnormal lactation, mammary adenocarcinoma
Gal	19	*GAL*	11 (q13.2)	*GAL*	29	47.913.700-47.928.200	*galanin*	abnormal branching of the mammary ductal tree, abnormal lactation
Birc4	X	*BIRC4*	X (q25)	*BIRC4*	X	6 M	*baculoviral IAP repeat-containing 4*	abnormal milk composition
Cited1	X	*CITED1*	X (q13.1)	*CITED1*	X	74 M	*cbp/p300-interacting transactivator with Glu/Asp-rich carboxy-terminal domain 1*	abnormal branching of the mammary ductal tree
Dkc1	X	*DKC1*	X (q28)	*DKC1*	X	40 M	*dyskeratosis congenita 1, dyskerin homolog (human)*	mammary gland tumor, mammary adenocarcinoma
Apmt1	15	NA	NA	NA	NA	NA	*accelerator of polyoma-induced mammary tumors 1*	mammary gland tumor
Apmt2	9	NA	NA	NA	NA	NA	*accelerator of polyoma-induced mammary tumors 2*	mammary gland tumor
Mtes	19	NA	NA	NA	NA	NA	*metastasis efficiency suppressor gene 1*	mammary gland tumor, mammary gland tumor
Mtv2	18	NA	NA	NA	NA	NA	*mammary tumor virus locus 2*	mammary alveolar hyperplasia, mammary gland tumor, mammary adenocarcinoma
Wap	11	NA	NA	NA	NA	NA	*whey acidic protein*	abnormal milk composition, mammary adenocarcinoma
Tp	4	NA	NA	NA	NA	NA	*taupe*	abnormal nipple morphology

NA, not available; M, location in mega base-pairs identified using bovine-human synteny map.
