# Supplementary material for: Database of cattle candidate genes and genetic markers for milk production and mastitis
Source: Anim Genet. 2009 Dec;40(6):832–51. doi: 10.1111/j.1365-2052.2009.01921.x (PMC2779988; doi:10.1111/j.1365-2052.2009.01921.x)
Supplement: Supplementary file 2 [file age0040-0832-SD2.pdf]

Table S2 Genes tested for association with milk and mastitis traits.

Mouse		Human homolog		Cattle homolog			Gene name	Reference
Gene	Chr.	Gene	Chr.	Gene	Chr.	Location (bp)		
Association-milk traits								
Pou1f1	16	*POU1F1*	3 (p11)	*POU1F1*	1	35,749,100-35,769,000	*POU class 1 homeobox 1*	Huang *et al.* 2008
Stat1	1	*STAT1*	2 (q32.2)	*STAT1*	2	83.359.000-83.440.000	*signal transducer and activator of transcription 1*	Cobanoglu *et al.* 2006
Lep	6	*LEP*	7 (q31.3)	*LEP*	4	95.665.000-95.707.000	*leptin*	Leifers *et al.* 2005<br>Banos *et al.* 2008<br>Chebel *et al.* 2008
Olr1	6	*OLR1*	12 (p13.2-p12.3)	*OLR1*	5	107.277.400 - 107.305.700	*oxidized low density lipoprotein (lectin-like) receptor 1*	Khatib *et al.* 2006
Ppargc1a	5	*PPARGC1A*	4 (p15.1)	*PPARGC1A*	6	44.652.000-44.919.000	*peroxisome proliferative activated receptor, gamma, coactivator 1 alpha*	Weikard *et al.* 2005
Abcg2	6	*ABCG2*	4 (q22)	*ABCG2*	6	37.298.000-37.475.000	*ATP-binding cassette, sub-family G (WHITE), member 2*	Olsen *et al.* 2007<br>Ron *et al.* 2006<br>Cohen-Zinder *et al.* 2005
Spp1	5	*SPP1*	4 (q21-q25)	*SPP1*	6	37.506.500-37.523.900	*secreted phosphoprotein 1 (osteopontin)*	Leonard *et al.* 2005
Csn1s1	5	*CSN1S1*	4 (q21.1)	*CSN1S1*	6	88.511.600-88.544.300	*casein alpha s1*	Sanders *et al.* 2006<br>Kuss *et al.* 2005<br>Prinzenberg *et al.* 2003
Csn3	5	*CSN3*	4 (q21.1)	*CSN3*	6	88.278.000-88.322.000	*casein kappa*	Kaminski *et al.* 2006<br>Robitaile *et al.* 2007<br>Kaminski *et al.* 2008
NA	NA	NA	NA	*LGB*	11	107.163.200 - 107.175.000	*lactoglobulin, beta*	Kuss *et al.* 2003<br>Ganai *et al.* 2008

Dgat1	15	*DGAT1*	8 (q24.3)	*DGAT1*	14	442.100-448.800	*diacylglycerol O-acyltransferase 1*	Kaminski *et al.* 2006<br>Grisart *et al.* 2002<br>Kaupe *et al.* 2007<br>Banos *et al.* 2008<br>Kaminski *et al.* 2008<br>Anton *et al.* 2008<br>Hradecka *et al.* 2008
Cyp11b1	15 (44.9 cM)	*CYP11B1*	8 (q21)	*CYP11B1*	14	1,294,000-1,303,600	*cytochrome P450, subfamily XI B, polypeptide 1*	Kaupe *et al.*, 2007
Fgf2	3 (19.3 cM)	FGF2	4 (q26-q27)	FGF2	17	36,954,000-36,961,600	*fibroblast growth factor 2 (basic)*	Wang *et al.* 2008
Nod2	8 (C3)	NOD2	16 (q21)	NOD2	18	18,141,400-18,148,000	*nucleotide-binding oligomerization domain containing 2*	Pant *et al.* 2007
Fasn	11	*FASN*	17 (q25)	*FASN*	19	49.659.250-49.662.020	*fatty acid synthase*	Morris *et al.* 2007
Stat5a	11	*STAT5A*	17 (q11.2)	*STAT5A*	19	43.713.000-43.764.000	*signal transducer and activator of transcription 5A*	Brym *et al.* 2004<br>Khatib *et al.* 2008
Gh	11	*GH1*	17 (q24.2)	*GH1*	19	52.158.000-52.203.000	*growth hormone*	Zhou *et al.* 2005
Ccl2	11 (46.5 cM)	CCL2	17 (q11.2-q12)	CCL2	19	15,182,340-15,187,010	*chemokine (C-C motif) ligand 2*	Leyva-Baca *et al.* 2007
Ghr	15	*GHR*	5 (p13-p12)	*GHR*	20	33.770.000-34.200.000	*growth hormone receptor*	Blott *et al.* 2003<br>Kaminski *et al.* 2006<br>Kaminski *et al.* 2008<br>Hradecka *et al*, 2008
Serpina1	12	*SERPINA1*	14 (q32.1)	*SERPINA1*	21	59.296.400-59.319.900	*serine protease inhibitor*	Khatib *et al.* 2005
Ltf	9	*LTF*	3 (q21-q23)	*LTF*	22	54.289.000-54.372.000	*lactoferrin*	Kaminski *et al.* 2006<br>Kaminski *et al.* 2008
Prl	13	*PRL*	6 (p22.2-p21.3)	*PRL*	23	35.566.300-35.587.800	*prolactin*	Brym *et al.* 2005<br>He *et al.* 2006

NA	NA	NA	NA	*BoLA-DRB3*	23	40 M	*major histocompatibility complex, class II, DRB3*	Sharif *et al.* 1999<br>do Nascimento *et al.* 2006<br>Rupp *et al.* 2007
Scd2	19 (43.0 cM)	*SCD*	10 (q24.31)	*SCD*	26	20M	*stearoyl-CoA desaturase (delta-9-desaturase)*	Macciotta *et al.* 2008
Association-mastitis traits								
Il8rb	1	*IL8RB*	2 (q35)	*IL8RB*	2	110.580.400-110.599.100	*interleukin 8 receptor, beta*	Youngermann *et al.* 2004
Il8ra	1	*IL8RA*	2 (q35)	*IL8RA*	2	110.614.800-110.619.100	*interleukin 8 receptor, alpha*	Rambeaud and Pighetti 2007<br>Leyva-Bacca *et al.* 2008
Tlr4	4	*TLR4*	9 (q32-q33)	*TLR4*	8	112.418.500-112.446.100	*toll-like receptor 4*	Sharma *et al.* 2006b<br>Wang *et al.* 2007
Fezf2	14	*FEZF2*	3 (p14.2)	*FEZF2*	22	39.843.600-39.855.300	*fez family zinc finger 2*	Sugimoto *et al.* 2006
Ltf	9	*LTF*	3 (q21-q23)	*LTF*	22	54.289.000-54.372.000	*lactoferrin*	Wojdak-Maksimyec *et al.* 2006
NA	NA	NA	NA	*BoLA-DRB3*	23	26.307.000-26.382.000	*major histocompatibility complex, class II, DRB3*	Sharif *et al.* 1998<br>do Nascimento *et al.* 2006<br>Rupp *et al.* 2007
Fgf2	3 (19.3 cM)	*FGF2*	4 (q26-q27)	*FGF2*	17	36.954.000-36.961.600	*fibroblast growth factor 2 (basic)*	Wang *et al.* 2008
Nod2	8 (C3)	*NOD2*	16 (q21)	*NOD2*	18	18.141.400-18.148.000	*nucleotide-binding oligomerization domain containing 2*	Pant *et al.* 2007
Ccr2	9 (71.9 cM)	*CCR2*	3 (p21.31)	*CCR2*	22	54.404.230-54.405.620	*chemokine (C-C motif) receptor 2*	Leyva-Baca *et al.* 2007
Cyp11b1	15 (44.9 cM)	*CYP11B1*	8 (q21)	*CYP11B1*	14	1.294.000-1.303.600	*cytochrome P450, subfamily XI B, polypeptide 1*	Kaupe *et al.* 2007

NA, not available; M, location in mega base-pairs identified using bovine-human synteny map.
