# Supplementary material for: Database of cattle candidate genes and genetic markers for milk production and mastitis
Source: Anim Genet. 2009 Dec;40(6):832–51. doi: 10.1111/j.1365-2052.2009.01921.x (PMC2779988; doi:10.1111/j.1365-2052.2009.01921.x)
Supplement: Supplementary file 3 [file age0040-0832-SD3.pdf]

Table S3 Genes with expression patterns associated with mastitis.

Mouse		Human		Cattle			Gene name	Pathogen	Model org.	Reference
Gene	Chr.	Gene	Chr.	Gene	Chr.	Location (bp)				
Nfkbiz	16	*NFKBIZ*	3 (p12-q12)	*NFKBIZ*	1	47.287.100-47.314.600	*nuclear factor of kappa light polypeptide gene enhancer in B-cells inhibitor, zeta*	*E. coli*	mouse	Zheng *et al.* 2006
Ets2	16	*ETS2*	21 (q22.2)	*ETS2*	1	154.594.100-154.622.300	*E26 avian leukemia oncogene 2, 3' domain*	*E. coli*	mouse	Zheng *et al.* 2006
Ehhadh	16	*EHHADH*	3 (q26.3-q28)	*EHHADH*	1	83.709.000-83.846.000	*enoyl-Coenzyme A, hydratase/3-hydroxyacyl Coenzyme A dehydrogenase*	*E. coli*	mouse	Zheng *et al.* 2006
Ccl20	1	*CCL20*	2 (q33-q37)	*CCL20*	2	120.588.800-120.596.500	*chemokine (C-C motif) ligand 20*	*E. coli*	mouse	Zheng *et al.* 2006
Rbms1	2	*RBMS1*	2 (q24.2)	*RBMS1*	2	36.940.000-37.480.000	*RNA binding motif, single stranded interacting protein 1*	*E. coli*	mouse	Zheng *et al.* 2006
Ly75	2	*LY75*	2 (q24)	*LY75*	2	37.390.000-38.150.000	*lymphocyte antigen 75*	ND	cattle	Schwerin *et al.* 2003
S100a8	3	*S100A8*	1 (q21)	*S100A8*	3	18.480.300-18.485.100	*S100 calcium binding protein A8 (calgranulin A)*	*E. coli*	mouse	Zheng *et al.* 2006
Jun	4	*JUN*	1 (p32-p31)	*JUN*	3	21.663.000-21.674.600	*Jun oncogene*	*E. coli*	mouse	Zheng *et al.* 2006
Mcl1	3	*MCL1*	1 (q21)	*MCL1*	3	93.617.300-93.622.400	*myeloid cell leukemia sequence 1*	*E. coli*	mouse	Zheng *et al.* 2006
Il-6	5	*IL6*	7 (p21)	*IL6*	4	32.753.300-32.764.200	*interleukin 6*	*E. coli*<br>*E. coli*	mouse<br>cattle	Zheng *et al.* 2006<br>Pareek *et al.* 2005
Hgf	5	*HGF*	7 (q21.1)	*HGF*	4	40.160.000-40.550.000	*hepatocyte growth factor*	*E. coli*	mouse	Zheng *et al.* 2006
Btg1	10	*BTG1*	12 (q22)	*BTG1*	5	24.782.700-24.789.500	*B-cell translocation gene 1, anti-proliferative*	*E. coli*	mouse	Zheng *et al.* 2006
Etv6	6	*ETV6*	12 (p13)	*ETV6*	5	105.060.000-105.470.000	*ets variant gene 6 (TEL oncogene)*	*E. coli*	mouse	Zheng *et al.* 2006
Maff	15	*MAFF*	22 (q13.1)	*MAFF*	5	117.027.100-117.049.400	*v-maf musculoaponeurotic fibrosarcoma oncogene family, protein F (avian)*	*E. coli*	mouse	Zheng *et al.* 2006
Bid	6	*BID*	22 (q11.1)	*BID*	5	49.347.600-	*BH3 interacting domain death*	*E. coli*	mouse	Zheng *et al.* 2006

						49.359.700	*agonist*			
Gpd1	15	*GPD1*	12 (q12-q13)	*GPD1*	5	116.144.000-116.188.000	*glycerol-3-phosphate dehydrogenase 1 (soluble)*	*E. coli*	mouse	Zheng *et al.* 2006
Ifng	10	*IFNG*	12 (q14)	*IFNG*	5	32.794.400-32.808.400	*interferone gamma*	*E coli*<br>*K. pneumoniae*<br>*E. coli* and *S. aureus*	cattle<br>cattle<br>cattle	Lee *et al.* 2006<br>Bannerman *et al.* 2004a<br>Bannerman *et al.* 2004b
Cxcl5	5	*CXCL6*	4 (q21)	*CXCL5*	6	91.876.300-91.881.600	*chemokine (C-X-C motif) ligand 5*	*E. coli*	cattle	Pareek *et al.* 2005
Cxcl2	5	*CXCL2*	4 (q21)	*CXCL2*	6	23.746.000-23.986.000	*chemokine (C-X-C motif) ligand 2*	*E. coli*	mouse	Zheng *et al.* 2006
Cxcl1	5	*CXCL1*	4 (q21)	*CXCL1*	6	91.925.900-91.931.100	*chemokine (C-X-C motif) ligand 1*	*E. coli*	mouse	Zheng *et al.* 2006
Tacc3	5	*TACC3*	4 (p16.3)	*TACC3*	6	94.127.300-94.133.200	*transforming, acidic coiled-coil containing protein 3*	*E. coli*	mouse	Zheng *et al.* 2006
Nfkb1	3	*NFKB1*	4 (q24)	*NFKB1*	6	118.766.900-118.790.000	*nuclear factor of kappa light chain gene enhancer in B-cells 1, p105*	*E. coli*	cattle	Long *et al.* 2001
NA	NA	*IL8*	4q13-q21	*IL8*	6	91.787.600-91.797.000	*interleukin 8*	*E coli* and *S. aureus*<br>*K. pneumoniae*<br>*E. coli* and *S. aureus*	cattle<br>cattle<br>cattle	Lee *et al.* 2006<br>Bannerman *et al.* 2004a<br>Bannerman *et al.* 2004b
Irf1	11	*IRF1*	5 (q31.1)	*IRF1*	7	20.618.900-20.636.600	*interferon regulatory factor 1*	*E. coli*	mouse	Zheng *et al.* 2006
Cd14	18	*CD14*	5 (q31.1)	*CD14*	7	51.084.490-51.088.030	*CD14 antigen*	*E. coli.*<br>*Staphylococcus spp., yeast, coliforms*<br>*K. pneumoniae*<br>*E. coli* and *S. aureus*	mouse<br>cattle<br>cattle<br>cattle	Zheng *et al.* 2006<br>Lee *et al.* 2003<br>Bannerman *et al.* 2004a<br>Bannerman *et al.* 2004b
C3	17	*C3*	19 (p13.3-p13.2)	*C3*	7	16.291.000-16.381.000	*complement component 3*	*E. coli*	mouse	Zheng *et al.* 2006
Junb	8	*JUNB*	19 (p13.2)	*JUNB*	7	11.003.700-11.008.200	*Jun-B oncogene*	*E. coli*	mouse	Zheng *et al.* 2006
Gadd45b	10	*GADD45B*	19 (p13.3)	*GADD45B*	7	19.834.000-19.839.300	*growth arrest and DNA-damage-inducible 45 beta*	*E. coli*	mouse	Zheng *et al.* 2006

Map2k7	8	*MAP2K7*	19 (p13.3-p13.2)	*MAP2K7*	7	15.107.300-15.128.000	*mitogen activated protein kinase kinase 7*	*E. coli*	mouse	Zheng *et al.* 2006
Csf2	11	*CSF2*	5 (q31.1)	*CSF2*	7	21.081.100-21.087.000	*colony stimulating factor 2 (granulocyte-macrophage)*	*E coli* and *S. aureus*	cattle	Lee *et al.* 2006
Il12b	11	*IL12B*	5 (q31.1-q33.1)	*IL12B*	7	70.704.300-70.730.300	*interleukin 12b*	*E coli* and *S. aureus*	cattle	Lee *et al.* 2006
Tlr4	4	*TLR4*	9 (q32-q33)	*TLR4*	8	112.418.500-112.446.100	*toll-like receptor 4*	*E. coli*<br>ND	mouse<br>cattle	Zheng *et al.* 2006<br>Goldammer *et al.* 2004
Ostf1	19	*OSTF1*	9q13-q21	*OSTF1*	8	53.434.000-53.586.000	*osteoclast stimulating factor 1*	ND	cattle	Schwerin *et al.* 2003
Fyn	10	*FYN*	6 (q21)	*FYN*	9	40.320.000-40.700.000	*Fyn proto-oncogene*	*E. coli*	mouse	Zheng *et al.* 2006
Tnfaip3	10	*TNFAIP3*	6 (q23)	*TNFAIP3*	9	78.042.800-78.068.600	*tumor necrosis factor, alpha-induced protein 3*	*E. coli*	mouse	Zheng *et al.* 2006
B2m	2	*B2M*	15 (q21-q22.2)	*B2M*	10	106.122.900-106.138.500	*beta 2 microglobulin*	*E. coli*	cattle	Pareek *et al.* 2005
Fos	12	*FOS*	14 (q24.3)	*FOS*	10	88.957.300-88.965.900	*FBJ osteosarcoma oncogene*	*E. coli*	mouse	Zheng *et al.* 2006
Acot1	12	*ACOT1*	14 (q24)	*ACOT1*	10	87 M	*acyl-CoA thioesterase 1*	*E. coli*	mouse	Zheng *et al.* 2006
Rora	9	*RORA*	15 (q21-q22)	*RORA*	10	48.640.000-50.690.000	*RAR-related orphan receptor alpha*	ND	cattle	Schwerin *et al.* 2003
Birc2	9	*BIRC2*	11 (q22)	*BIRC2*	11	5 M	*baculoviral IAP repeat-containing 2*	*E. coli*	cattle	Pareek *et al.* 2005
Il1b	2	*IL1B*	2 (q14)	*IL1B*	11	48.242.900-48.263.900	*interleukin 1 beta*	*E. coli*<br>*E. coli*<br>*K. pneumoniae*<br>*E. coli* and *S. aureus*	mouse<br>cattle<br>cattle<br>cattle	Zheng *et al.* 2006<br>Pareek *et al.* 2005<br>Bannerman *et al.* 2004a<br>Bannerman *et al.* 2004b
Hadhb	5	*HADHB*	2 (p23)	*HADHB*	11	75.298.000-75.374.000	*hydroxyacyl-Coenzyme A dehydrogenase/3-ketoacyl-Coenzyme A thiolase/enoyl-Coenzyme A hydratase (trifunctional protein), beta subunit*	*E. coli*	mouse	Zheng *et al.* 2006
Vrk2	11	*VRK2*	2 (p16-p15)	*VRK2*	11	42.206.000-42.477.000	*vaccinia related kinase 2*	ND	cattle	Schwerin *et al.* 2003

Ptgs1	2	*PTGS1*	9 (q32-q33.3)	*PTGS1*	11	96.337.000-96.402.000	*prostaglandin-endoperoxide synthase 1*	ND	cattle	Pfaffl *et al.* 2003
Cdk8	5	*CDK8*	13 (q12)	*CDK8*	12	33.096.000-33.217.000	*cyclin-dependent kinase 8*	ND	cattle	Schwerin *et al.* 2003
Cebpb	2	*CEBPB*	20 (q13.1)	*CEBPB*	13	83M	*CCAAT/enhancer binding protein (C/EBP), beta*	*E. coli*	mouse	Zheng *et al.* 2006
Map3k8	18	*MAP3K8*	10 (p11.23)	*MAP3K8*	13	35.203.000-35.268.000	*mitogen activated protein kinase kinase kinase 8*	*E. coli*	mouse	Zheng *et al.* 2006
Mmp9	2	*MMP9*	20 (q11.2-q13.1)	*MMP9*	13	75.514.200-75.532.500	*matrix metallopeptidase 9*	*E. coli*	cattle	Long *et al.* 2001
Ahcy	2	*AHCY*	20 (cen-q13.1)	*AHCY*	13	64.337.780-64.340.670	*S-adenosylhomocysteine hydrolase*	ND	cattle	Schwerin *et al.* 2003
Lbp	2	*LBP*	20 (q11.23-q12)	*LBP*	13	67.761.000-67.869.000	*lipopolysaccharide binding protein*	*K. pneumoniae*<br>*E. coli* and *S. aureus*	cattle<br>cattle	Bannerman *et al.* 2004a<br>Bannerman *et al.* 2004b
Ly96	1	*LY96*	8 (q21.11)	*LY96*	14	54 M	*lymphocyte antigen 96*	*E. coli*	mouse	Zheng *et al.* 2006
Prkdc	16	*PRKDC*	8 (q11)	*PRKDC*	14	18.986.000-19.305.000	*protein kinase, DNA activated, catalytic polypeptide*	ND	cattle	Schwerin *et al.* 2003
Hspa8	9	*HSPA8*	11 (q24.1)	*HSPA8*	15	32.344.800-32.355.900	*heat shock protein 8*	*E. coli*	mouse	Zheng *et al.* 2006
Casp4	9	*CASP4*	11 (q22.2-q22.3)	*CASP4*	15	2.083.000-2.131.000	*caspase 4, apoptosis-related cysteine peptidase*	*E. coli*	mouse	Zheng *et al.* 2006
Birc3	9	*BIRC3*	11 (q22)	*BIRC3*	15	5.373.000-5.450.000	*baculoviral IAP repeat-containing 3*	*E. coli*	mouse	Zheng *et al.* 2006
Ucp3	7	*UCP3*	11 (q13)	*UCP3*	15	53.096.400-53.122.700	*uncoupling protein 3 (mitochondrial, proton carrier)*	*E. coli*	mouse	Zheng *et al.* 2006
Hnrpu	1	*HNRPU*	1 (q44)	*HNRPU*	16	29.695.100-29.699.100	*heterogeneous nuclear ribonucleoprotein U*	ND	cattle	Schwerin *et al.* 2003
Selp	1	*SELP*	1 (q22-q25)	*SELP*	16	34.635.000-34.743.000	*selectin, platelet*	*E. coli*	mouse	Zheng *et al.* 2006
Rsc1a1	NA	*RSC1A1*	1 (p36.1)	*RSC1A1*	16	49.123.000-49.212.000	*regulatory solute carrier protein, family 1, member 1*	ND	cattle	Schwerin *et al.* 2003
Ptgs2	1	*PTGS2*	1 (q25.2-q25.3)	*PTGS2*	16	65.506.300-65.525.400	*prostaglandin-endoperoxide synthase 2*	ND	cattle	Pfaffl *et al.* 2003
Tlr2	3	*TLR2*	4 (q32)	*TLR2*	17	4.271.600-4.304.700	*toll-like receptor 2*	E. coli<br>ND	mouse.<br>cattle	Zheng *et al.* 2006<br>Goldammer *et al.* 2004

Taok3	5	*TAOK3*	12 (q)	*TAOK3*	17	59.650.000-60.130.000	*TAO kinase 3*	ND	cattle	Schwerin *et al.* 2003
Hp	8	*HP*	16 (q22.1)	*HP*	18	38.128.500-38.143.900	*haptoglobin*	*E. coli*	mouse	Zheng *et al.* 2006
Relb	7	*RELB*	19 (q13.32)	*RELB*	18	52.460.000-52.527.000	*avian reticuloendotheliosis viral (v-rel) oncogene related B*	*E. coli*	mouse	Zheng *et al.* 2006
Kcnn4	7	*KCNN4*	19 (q13.2)	*KCNN4*	18	51.693.800-51.728.000	*potassium intermediate/small conductance calcium-activated channel, subfamily N, member 4*	*E. coli*	mouse	Zheng *et al.* 2006
Bax	7	*BAX*	19 (q13.3-q13.4)	*BAX*	18	55.349.200-55.359.700	*Bcl2-associated X protein*	*E. coli*	cattle	Long *et al.* 2001
C5ar1	7	*C5AR1*	19 (q13.3-q13.4)	*C5AR1*	18	54.091.000-54.138.000	*complement component 5a receptor 1*	*K. pneumoniae*<br>*E. coli* and *S. aureus*	cattle<br>cattle	Bannerman *et al.* 2004a<br>Bannerman *et al.* 2004b
Ccl5	11	*CCL5*	17 (q11.2-q12)	*CCL5*	19	13.965.800-13.982.700	*chemokine (C-C motif) ligand 5*	*E. coli*	cattle	Pareek *et al.* 2005
Stat3	11	*STAT3*	17 (q21.31)	*STAT3*	19	43.730.000-43.808.000	*signal transducer and activator of transcription 3*	*E. coli*	mouse	Zheng *et al.* 2006
Ccl2	11	*CCL2*	17 (q11.2-q12)	*CCL2*	19	13.820.900-13.824.700	*chemokine (C-C motif) ligand 2*	*E. coli*	mouse	Zheng *et al.* 2006
Ccl7	11	*CCL7*	17 (q11.2-q12)	*CCL7*	19	15.663.900-15.668.600	*chemokine (C-C motif) ligand 7*	*E. coli*	mouse	Zheng *et al.* 2006
Ccl3	11	*CCL3*	17 (q11-q21)	*CCL3*	19	10 M	*chemokine (C-C motif) ligand 3*	*E. coli*	mouse	Zheng *et al.* 2006
Cxcl16	11	*CXCL16*	17 (p13)	*CXCL16*	19	26.980.700-26.989.400	*chemokine (C-X-C motif) ligand 16*	*E. coli*	mouse	Zheng *et al.* 2006
Acly	11	*ACLY*	17 (q12-q21)	*ACLY*	19	43.374.000-43.485.000	*ATP citrate lyase*	*E. coli*	mouse	Zheng *et al.* 2006
Trp53	11	*TP53*	17 (p13.1)	*TP53*	19	27.870.400-27.901.300	*transformation related protein 53*	ND	cattle	Schwerin *et al.* 2003
Nr1d1	11	*NR1D1*	17 (q11.2)	*NR1D1*	19	41.649.900-41.668.200	*nuclear receptor subfamily 1, group D, member 1*	ND	cattle	Schwerin *et al.* 2003
Dusp1	17	*DUSP1*	5 (q34)	*DUSP1*	20	4.573.500-4.581.200	*dual specificity phosphatase 1*	*E. coli*	mouse	Zheng *et al.* 2006
Calm2	17	*CALM2*	2 (p21)	*CALM2*	20	8.507.370-8.510.270	*calmodulin 2*	*E. coli*	mouse	Zheng *et al.* 2006
Oxct1	15	*OXCT1*	5 (p13.1)	*OXCT1*	20	34.743.000-35.044.000	*3-oxoacid CoA transferase 1*	*E. coli*	mouse	Zheng *et al.* 2006
Nfkbia	12	*NFKBIA*	14 (q13)	*NFKBIA*	21	46.437.800-	*nuclear factor of kappa light*	*E. coli*	mouse	Zheng *et al.* 2006

						46.446.300	*chain gene enhancer in B-cells inhibitor, alpha*			
Bcl2a1a	9	*BCL2A1*	15 (q24.3)	*BCL2A1*	21	25.747.600-25.772.000	*B-cell leukemia/lymphoma 2 related protein A1a*	*E. coli*	mouse	Zheng *et al.* 2006
Fezf2	14	*FEZF2*	3 (p14.2)	*FEZF2*	22	39.843.600-39.855.300	*Fez family zinc finger 2*	*E. coli*	cattle	Sugimoto *et al.* 2006
Ltf	9	*LTF*	3 (q21-q23)	*LTF*	22	54.289.000-54.372.000	*lactoferrin*	ND	cattle	Pfaffl *et al.* 2003
Dcdc2a	13	*DCDC2*	6 (p22.1)	*DCDC2*	23	34 M	*doublecortin domain containing 2*	*E. coli*	cattle	Pareek *et al.* 2005
Cfb	17	*CFB*	6 (p21.3)	*CFB*	23	27.202.700-27.217.800	*complement factor B*	*E. coli*	mouse	Zheng *et al.* 2006
Kcnk5	14	*KCNK5*	6 (p21)	*KCNK5*	23	13.528.000-13.625.000	*potassium channel, subfamily K, member 5*	*E. coli*	mouse	Zheng *et al.* 2006
Ssr1	13	*SSR1*	6 (p24.3)	*SSR1*	23	48.904.000-48.950.000	*signal sequence receptor, alpha*	*S. agalactiae*	cattle	Schwerin *et al.* 2003
Tnf	17	*TNF*	6 (p21.3)	*TNF*	23	27.529.200-27.536.100	*tumor necrosis factor*	*E coli* and *S. aureus*<br>*K. pneumoniae*<br>*E. coli* and *S. aureus*<br>ND	cattle<br>cattle<br>cattle<br>cattle	Lee *et al.* 2006<br>Bannerman *et al.* 2004a<br>Bannerman *et al.* 2004b<br>Pfaffl *et al.* 2003
Lyn	4	*LYN*	8 (q13)	*YES1*	24	36.749.000-36.911.000	*Yamaguchi sarcoma viral (v-yes-1) oncogene homolog*	*E. coli*	mouse	Zheng *et al.* 2006
Bcl2	1	*BCL2*	18 (q21.3)	*BCL2*	24	63.860.000-64.370.000	*B-cell leukemia/lymphoma 2*	*E. coli*	cattle	Long *et al.* 2001
Actb	5	*ACTB*	7 (p15-p12)	*ACTB*	25	40.632.600-40.641.100	*actin, beta, cytoplasmic*	*E coli* and *S. aureus*	cattle	Lee *et al.* 2006
Plce1	19	*PLCE1*	10 (q23)	*PLCE1*	26	15.580.000-16.500.000	*phospholipase C, epsilon 1*	*S. agalactiae*	cattle	Schwerin *et al.* 2003
NA	NA	NA	NA	*LAP*	27	7.407.100-7.411.600	*lingual antimicrobial peptide*	*S. uberis, S. aureus, Cornybacterium spp.*	cattle	Swanson *et al.* 2004
Plat	8	*PLAT*	8 (p12)	*PLAT*	27	39.451.000-39.511.000	*plasminogen activator, tissue*	*E. coli*	cattle	Pareek *et al.* 2005
Acsl1	8	*ACSL1*	4 (q34-q35)	*ACSL1*	27	16.445.000-16.607.000	*acyl-CoA synthetase long-chain family member 1*	*E. coli*	mouse	Zheng *et al.* 2006
Kcnk1	8	*KCNK1*	1 (q42-q43)	*KCNK1*	28	4.743.000-4.911.000	*potassium channel, subfamily K, member 1*	*E. coli*	mouse	Zheng *et al.* 2006

Alox5	6	*ALOX5*	10 (q11.2)	*ALOX5*	28	44.440.000-44.556.000	*arachidonate 5-lipoxygenase*	ND	cattle	Pfaffl *et al.* 2003
Rela	19	*RELA*	11 (q13)	*RELA*	29	45.686.400-45.708.100	*v-rel reticuloendotheliosis viral oncogene homolog A (avian)*	*E. coli*	mouse	Zheng *et al.* 2006
Saa2	7	*SAA2*	11 (p15.1-p14)	*SAA2*	29	22 M	*serum amyloid A 2*	*E. coli*	mouse	Zheng *et al.* 2006
Saa1	7	*SAA1*	11 (p15.1)	*SAA1*	29	22 M	*serum amyloid A 1*	*E. coli*	mouse	Zheng *et al.* 2006
Saa3	7	*SAA3*	11 (p15.1)	*SAA3*	29	27.681.500-27.690.900	*serum amyloid A 3*	*E. coli*<br>ND	mouse<br>cattle	Zheng *et al.* 2006<br>Schwerin *et al.* 2003
Tirap	9	*TIRAP*	11 (q24.2)	*TIRAP*	29	31.225.600-31.237.700	*toll-interleukin 1 receptor (TIR) domain-containing adaptor protein*	*E. coli*	mouse	Zheng *et al.* 2006
Ahnak	19	*AHNAK*	11 (q12.2)	*AHNAK*	29	42.566.300-42.578.700	*AHNAK nucleoprotein (desmoyokin)*	*S. agalactiae*	cattle	Schwerin *et al.* 2003
Cdkl5	X	*CDKL5*	X (p22)	*CDKL5*	X	15 M	*serine/threonine kinase 9 (cyclin-dependent kinase-like 5)*	*S. agalactiae*	cattle	Schwerin *et al.* 2003
Enox2	X	*ENOX2*	X (q25-q26.2)	*ENOX2*	X	80 M	*ecto-NOX disulfide-thiol exchanger 2*	ND	cattle	Schwerin *et al.* 2003
Defb5	8	NA	NA	*BNBD5*	NA	UN	*defensin beta 5*	ND	cattle	Goldammer *et al.* 2004

NA, not available; ND, not defined; M, location in mega base-pairs identified using bovine-human synteny map.
