# Supplementary material for: Database of cattle candidate genes and genetic markers for milk production and mastitis
Source: Anim Genet. 2009 Dec;40(6):832–51. doi: 10.1111/j.1365-2052.2009.01921.x (PMC2779988; doi:10.1111/j.1365-2052.2009.01921.x)
Supplement: Supplementary file 4 [file age0040-0832-SD4.pdf]

Table S4. Milk protein genes and their variants (adapted from Farrell *et al.* 2004 and Buchberger & Dovc 2000).

Gene	Chr.	Location	Gene description	Milk protein	Number of variants	Protein variants
LALBA	5 (q21)	34.385.300-34.390.400	*lactalbumin, alpha*	α - lactalbumin	3	A, B*, C
CSN1S1	6 (q26-q33)	88.294.650-88.295.570	*casein alpha s1*	α_{S1} - casein	11	A, B*, C*, D, E, F, G,H, X, X', Y
CSN1S2	6 (q31)	88.395.000-88.441.000	*casein alpha s2*	α_{S2} - casein	4	A*, B, C, D
CSN2	6 (q31)	88.322.000-88.345.400	*casein beta*	β - casein	17	A^1*, A^2*, A^3, $A^3_{Mongolie}$, A^4, A^5, B*, B_2, B_z, C, D, E, F, G, H^1, H^2, I
CSN3	6 (q31)	88.511.600-88.544.300	*casein kappa*	κ - casein	16	A*, A^1 , A_{1SS}, B*, B^2, C, E, F^1, F^2, G^1, G^2, H, I, J, X, Y
LGB	11 (q28)	107.163.200-107.175.000	*lactoglobulin, beta*	β - lactoglobulin	16	A*, B*, C, D, Dr, D_{Yak}, E, F, G, H, I, J, W, X, Y, Z
IGHG1	21 (q24)	60 M	*immunoglobulin heavy constant gamma 1*	immunoglobulin G1	2	$IgG1^a$, $IgG1^b$
IGHG2	21(q23-q24)	60 M	*immunoglobulin heavy constant gamma 2*	immunoglobulin G2	2	$IgG2^a$, $IgG2^b$
IGHG3	21 (q24)	60 M	*immunoglobulin heavy constant gamma 3*	immunoglobulin G3	2	$IgG3^a$, $IgG3^b$

*, common variants; M, location in mega base-pairs identified using bovine-human synteny map.
