# Supplementary material for: Database of cattle candidate genes and genetic markers for milk production and mastitis
Source: Anim Genet. 2009 Dec;40(6):832–51. doi: 10.1111/j.1365-2052.2009.01921.x (PMC2779988; doi:10.1111/j.1365-2052.2009.01921.x)
Supplement: Supplementary file 5 [file age0040-0832-SD5.pdf]

Table S5 miRNAs expressed in mammary gland (adapted from Gu *et al.* 2007).

miRNA	Chr.	Location (bp)	Sequence	Overlapping QTL	Putative miRNA target sites in candidate genes
bta-miR-15b	1	108.876.544-108.876.641	UAGCAGCACAUCAUGGUUUACA	PY, MY	*LALBA, CHEK1, TNFAIP3, CSN1S2, PIN1, PTHLH, MIA, SLC39A8, BTN1A1, SSR2, APOD*
bta-mir-16	1	108.876.401-108.876.493	UAGCAGCACGUAAAUAUUGGC	PY, MY	*LALBA, CHEK1, UCP3, BIN1, ELF2, CXCL2, ETS2, PTHLH, NBR1, ADFP, EGF, BTN1A1, KRTCAP2, APOD*
bta-mir-29a	4	97.826.811-97.826.874	CUAGCACCAUCUGAAAUCGGUUA		*XDH, OXTR, MEOX2, FOS, B2M, SLC1A4, IGFALS, MYL6, GPS1, FABP3, EGF, SL39A8*
bta-mir-148b	5	28.577.700-28.577.789	UCAGUGCAUCACAGAACUUUGU	MY, PC	*STAT5A, NCOA1, VGF, BCL2A1, CCL3, CBL, OSTF1, GUSB, RINT1, HOXC6, NIT1, CSF1, RAB20, ACTA2, DVL2, DPEP3, DHRS1, PFKL*
bta-mir-26a	5	60.085.852-60.085.935	UUCAAGUAAUCCAGGAUAGGCU	FY, PY	*CSN3, KCNK1, CDK8, IFNG, HOXC6, UCP3, HSPA8, HGF, CFB, GADD45B*
bta-mir-145	7	60.270.231-60.270.318	GUCCAGUUUUCCCAGGAAUCCCU	SCS, MY, PY, FP	*YES1, TIRAP, LBP, C5AR1, RINT1*
bta-mir-23a	7	10.102.128-10.102.200	AUCACAUUGCCAGGGAUUUCC	FY, PY	*PTGS1, CSN2, YES1, LATS1, STAT5B, VRK2, IL6, AEBP1, NEO1, KCNK5, TNF*
bta-mir-101	8	41.898.819-41.898.897	UACAGUACUGUGAUAACUGAA	MSPD	*CHEK1, GJA1, CDK8, BCL2A1, LBP, PROKR2, HSPA8, CBL, PGR, JAK2, JUNB, FOS, GLI3, HOXC6, CXCL1, OXTR, CDH3, DUSP1, TNFSF11, MYL6, RAB3IP, CSRP2, STMN1*
bta-mir-23b	8	85.962.044-85.962.103	AUCACAUUGCCAGGGAUUACCAC	PP	*PTGS1, YES1, LATS1, STST5B, VRK2, KCNK5, AEBP1, IL6, CXCL5, VRK2, LEP, CTSC, GK, DVL2, HSPD1*
bta-let-7a	8	89.743.299-89.743.378	UGAGGUAGUAGGUUGUAUAGUU		*LALBA, TP53, CDK8, MFGE8, MKL1, HOXC6, RSC1A1, PROKR2, NTN1, CD14, CFB, IL6, CSN3, ELF2, SRPR, QSOX1, LLGL2, SLC35B1, CAPN6, NUCB2, TAGLN, NANS*

bta-let-7f	8	89.743.299-89.743.378	UGAGGUAGUAGAUUGUAUAGUU		*LALBA, TP53, CDK8, MKL1, HOXC6, RSC1A1, NTN1, ELF2, CFB, IL6, CSN3, SRPR, QSOX1, LLGL2, SLC35B1, CAPN6, NUCB2, DHRS1, CD320*
bta-mir-126	11	108.009.392-108.009.464	CGUACCGUGAGUAAUAAUGCG		*TIRAP, SELP, CXCL16, MNT, ELF2. ACTA2, NUCB2*
bta-mir-199b	11	102419001-102419100	CCCAGUGUUUAGACUAUCUGUUC		*CSN3, PTGS1, NRG3, NTN1, CXCL16, STAT1, DUSP1, GUSB, NEO1, DDR1, JUNB, CSF2, MAP3K11, CSRP2, PIGR, CYB561, ENO3*
bta-mir-15a	12	18.887.743-18.887.825	UAGCAGCACAUAAUGGUUUGU	MY, PY, PP, FY	*LALBA, CHEK1, HR, DDR1, CSN1S2, KCNN4,PTHLH, NBR1*
bta-mir-30b	14	6.477.866-6.477.953	UGUAAACAUCCUACACUCAGCU	MY, PP, FY, FP	*SIRT1, AHNAK, NME1, CCL3, UCP3, MSX2, IL6ST, HOXC6, RAB20, CMA1, RAB3IP, ACTA2, CYB561, NME1, GLDC, FOXH1*
bta-mir-125b	15	31.406.340-31.406.427	UCCCUGAGACCCUAACUUGUGA	SCS	*TNF, KCNK5, B2M, BAX, OXCT1, VGF, TNFAIP3, NFKBIA, ERBB3, IFNG, MAP3K11, TNXB, IRF4, GNA15, ELOVL1, PFKL*
bta-mir-199a	16	36.247.788-36.247.891	ACAGUAGUCUGCACAUUGGUUA		*PLG, MAP3K11, CSRP2, LLGL2*
bta-mir-205	16	72.083.257-72.083.325	UCCUUCAUUCCACCGGAGUCUG		*CSF1R, INHBB, SLC6A3, KCNN4, ROGDI*
bta-mir-214	16	36.241.995-36.242.104	GUACAGCAGGCACAGACAGGCAGU		*LALBA, MFGE8, HOXC6, SELP, CFB, APM1, LTF, MAP3K11, COX8, FAM110A, NBR1, LCN2, TCN2, LAMB3, EGF, BTN1A1, CTSS, APOD, ST6GAL1*
bta-mir-29c	16	73.887.217-73.887.304	UAGCACCAUUUGAAAUCGGUUA		*ID2, BRCA2, OXCT1, FOS, NIT1, EHHADH, RBM9, MYL6, TFAP2C, CA2, ITGAV*
bta-mir-195	19	27.179.312-27.179.398	UAGCAGCACAGAAAUAUUGGCA	SCS, FY	*LALBA, CHEK1, IL6ST, ELF2, NFKB1, DDR1, SSR1, PTHLH, NBR1, EGF, BTN1A1, KRTCAP2*
bta-mir-142	19	8.516.953-8.517.039	CCCAUAAAGUAGAAAGCACUA		*CSN2, LBP, CDKL5, C5AR1, PPARG, NEO1, RORA, B2M, CD9, ITGAV*
bta-mir-142b	19	1.061.191-1.061.105	CAUAAAGUAGAAAGCACUACUA		*CSN2, LBP, CDKL5, C5AR1, PPARG1, NEO1, RORA, B2M, CD9, ITGAV, TMEM165*
bta-mir-21	19	10.049.133-10.049.204	UAGCUUAUCAGACUGAUGUUGACU		*PPYR1, GLI2, C5AR1, B2M, DCDC2, NR3C1, CSRP2, MKI67*

bta-mir-497	19	27.179.621-27.179.732	CAGCAGCACACUGUGGUUUGUA	SCS, FY	*LALBA, CHEK1, TNFAIP3, ACSL1, KCNN4, RINT1, PTHLH,*
bta-mir-103	20	2.864.127-2.864.198	AGCAGCAUUGUACAGGGCUAUGA	PY, FY	*CHEK1, PLG, TIMP3, ACLY, CCL3, HOXD9, VRK2, SEPX1, PEX14*
bta-mir-342	21	65.160.823-65.160.916	UCUCACACAGAAAUCGCACCCAUCU		*CDKL5, PTCH1, MYBL1, DUSP1, SLC6A3, CDH3, KCNK5, GADD45B, CTBP2, QSOX1*
bta-mir-191	22	51.902.736-51.902.826	CAACGGAAUCCCAAAAGCAGCUG	EY, PP, FY	*LALBA, XDH, HOXD9, TP73L, CD9, MGP, FOLR1, FABP3, IRX3, GNA15*
bta-mir-374	X	46.871.549-46.871.620	UUAUAAUACAACCUGAUAAGUG		*BIRC2, LATS1, PTGS2, HSPA8, ZFPM2, RBMS1, MSX1, HOXC6, NR3C1, PYCR2*
bta-mir-210	NA	NA	ACUGUGCGUGUGACAGCGGCUGA		*NCOA1, YES1, HOXC6, PROKR2, FASN, TBX2, GUSB, NME1, AEBP1, ROGDI*
bta-mir-143	NA	NA	UGAGAUGAAGCACUGUAGCUC		NA
bta-mir-339	NA	NA	UACCUGUCCUCCAGGAGCUCA		NA

NA, not available; FY, fat yield; MY, milk yield; CM, clinical mastitis; SCS, somatic cell score; PY, protein yield; FP, fat percentage; PP, protein percentage; EY, energy yield.
